# High-throughput identification and rational design of synergistic small-molecule pairs for combating and bypassing antibiotic resistance

**DOI:** 10.1371/journal.pbio.2001644

**Published:** 2017-06-20

**Authors:** Morgan A. Wambaugh, Viplendra P. S. Shakya, Adam J. Lewis, Matthew A. Mulvey, Jessica C. S. Brown

**Affiliations:** Division of Microbiology and Immunology, Pathology Department, University of Utah School of Medicine, Salt Lake City, Utah, United States of America; Pennsylvania State University, United States of America

## Abstract

Antibiotic-resistant infections kill approximately 23,000 people and cost $20,000,000,000 each year in the United States alone despite the widespread use of small-molecule antimicrobial combination therapy. Antibiotic combinations typically have an additive effect: the efficacy of the combination matches the sum of the efficacies of each antibiotic when used alone. Small molecules can also act synergistically when the efficacy of the combination is greater than the additive efficacy. However, synergistic combinations are rare and have been historically difficult to identify. High-throughput identification of synergistic pairs is limited by the scale of potential combinations: a modest collection of 1,000 small molecules involves 1 million pairwise combinations. Here, we describe a high-throughput method for rapid identification of synergistic small-molecule pairs, the overlap^2^ method (O2M). O2M extracts patterns from chemical-genetic datasets, which are created when a collection of mutants is grown in the presence of hundreds of different small molecules, producing a precise set of phenotypes induced by each small molecule across the mutant set. The identification of mutants that show the same phenotype when treated with known synergistic molecules allows us to pinpoint additional molecule combinations that also act synergistically. As a proof of concept, we focus on combinations with the antibiotics trimethoprim and sulfamethizole, which had been standard treatment against urinary tract infections until widespread resistance decreased efficacy. Using O2M, we screened a library of 2,000 small molecules and identified several that synergize with the antibiotic trimethoprim and/or sulfamethizole. The most potent of these synergistic interactions is with the antiviral drug azidothymidine (AZT). We then demonstrate that understanding the molecular mechanism underlying small-molecule synergistic interactions allows the rational design of additional combinations that bypass drug resistance. Trimethoprim and sulfamethizole are both folate biosynthesis inhibitors. We find that this activity disrupts nucleotide homeostasis, which blocks DNA replication in the presence of AZT. Building on these data, we show that other small molecules that disrupt nucleotide homeostasis through other mechanisms (hydroxyurea and floxuridine) also act synergistically with AZT. These novel combinations inhibit the growth and virulence of trimethoprim-resistant clinical *Escherichia coli* and *Klebsiella pneumoniae* isolates, suggesting that they may be able to be rapidly advanced into clinical use. In sum, we present a generalizable method to screen for novel synergistic combinations, to identify particular mechanisms resulting in synergy, and to use the mechanistic knowledge to rationally design new combinations that bypass drug resistance.

## Author summary

Antibiotic resistance is a growing problem that threatens our ability to treat systemic bacterial infections. One strategy to combat antibiotic resistance is the use of synergistic antibiotic pairs that, when combined, have activity that is considerably greater than the sum of each individual drug’s activity on its own. Synergistic combinations can even inhibit the growth of bacteria that are resistant to the individual treatment drugs. However, synergistic pairs are rare and difficult to identify. High-throughput identification of synergistic pairs is challenging due to scale: 1 million different pairs are possible for a relatively small collection of 1,000 small molecules. Here, we describe a high-throughput method for rapid identification of synergistic small-molecule pairs, termed the overlap^2^ method (O2M), that dramatically speeds up the screening process. First, we identify mutants that show the same phenotype when treated with each individual molecule in a synergistic pair, then use this information to guide screens for additional synergistic pairs. As a proof of concept, we studied the synergistic antibiotic pair trimethoprim and sulfamethizole, and we identified several additional synergistic molecules. Among these is the antiviral drug azidothymidine (AZT), which blocks bacterial DNA replication. Trimethoprim and sulfamethizole both inhibit folate biosynthesis, which is necessary for the proper synthesis of nucleotides for DNA replication and repair. We found that reduced nucleotide levels sensitize *E*. *coli* cells to AZT. When we substitute trimethoprim with other small molecules that also reduce nucleotide levels, we find that these small molecules also act synergistically with AZT. Indeed, AZT in combination with trimethoprim substitutes inhibits the growth of trimethoprim-resistant clinical isolates more potently than trimethoprim and AZT or trimethoprim and sulfamethizole. This work demonstrates that when we resolve the pathways that underlie synergistic interactions, we can then identify additional small molecules that act by similar mechanisms, providing a means to bypass antibiotic resistance.

## Introduction

Small-molecule antimicrobial therapy facilitated one of the greatest increases in lifespan in history but is endangered by the rise of antimicrobial-resistant “superbugs” [1]. The CDC estimates that antibiotic-resistant bacteria cause more than 2 million infections and 23,000 deaths annually in the United States alone [2]. Combating antibiotic resistance requires a regular supply of new antimicrobial drugs, as bacteria inevitably acquire resistance to any single drug. Two main approaches are commonly used to identify additional antibiotics: new drug discovery and repurposing of drugs already approved for other conditions [3–6]. New drugs are more likely to result in breakthroughs but require a large upfront capital investment in time-consuming clinical trials. Repurposing can move drugs into the clinic without extensive trials but will not identify novel drug classes or structures [7].

This study explores a third strategy to combat antimicrobial resistance: synergistic combination therapy. Synergy occurs when 2 drugs act together with efficacy beyond the additive effect of each drug on its own [8]. Since synergistic drug pairs can kill microbes that are resistant to 1 drug in the pair [9] and are thought to slow the evolution of resistance [10,11], they have generated considerable interest as a promising way to overcome antimicrobial drug resistance.

Delays in the commencement of treatment of severe infections can dramatically increase mortality rates—for example, septic patients face an 8% increase in mortality for each hour’s delay [12]. Therefore, combinations of antimicrobials are commonly used prior to the identification of the causal organism. Most of the combinations currently employed are additive, but meta-analyses of clinical trials indicate better outcomes if synergistic combinations are used when the causal organism is unknown [12–15]. Molecules in additive combinations also frequently act against the same target or target pathways [16] and thus are potentially more susceptible to resistance-conferring mutation than combinations with different targets. However, few synergistic combinations have been identified [17] (S1 Table), and high-throughput identification has been challenging due to the numbers involved: a collection of 1,000 molecules has 1 million potential pairwise combinations.

We previously described a new approach to high-throughput identification of synergistic small-molecule pairs: the overlap^2^ method (O2M) [18]. O2M uses at least 1 known synergistic interaction to predict many additional interactions from large-scale chemical-genetics data. The rationale was that each small molecule in a synergistic pair produces a set of phenotypes—chemical-genetic signature—in a precise set of mutants that show reduced or enhanced growth in the presence of each molecule. When mutants exhibit the same phenotype when treated with known synergistic molecules, we predict that any molecule that induces the same phenotype from the same mutant will act synergistically with each original synergistic molecule. This was indeed the case, even when the known synergistic molecules have different mechanisms of action [18]. When we validated O2M on the pathogenic fungus *Cryptococcus neoformans*, we identified 36 new synergistic interactions with a low false positive rate (73% of the predictions were verified) [18]. Since then, several other groups published methods using chemical-genetics datasets to identify synergistic small-molecule interactions [19–21]. Chemical-genetics datasets are widely available for a variety of pathogenic microbes, including *Plasmodium falciparum* [22,23], *Mycobacterium tuberculosis* [24], *Candida albicans* [25,26], *Candida glabrata* [27], and *C*. *neoformans* [18]. Thus, methods identifying synergistic drug interactions from chemical-genetics datasets are potentially broadly applicable.

In this study, we show that O2M is also applicable to bacterial pathogens and antibiotics. Furthermore, we expand its utility with a novel high-throughput screening method for synergistic combinations and elucidate the molecular mechanism of a new drug combination. From this, we go on to rationally design synergistic combinations with different targets but the same phenotypic consequences, thus bypassing the original resistance mechanism. Indeed, our rationally designed synergistic combinations efficiently inhibit growth of clinical isolates resistant to the original antibiotic combination. In sum, we have developed an adaptable method for high-throughput screening for synergistic small-molecule pairs that facilitates rational design of synergistic small-molecule combinations, thereby addressing a key medical need in the treatment of drug-resistant infections.

## Results

### O2M uses a chemical-genetics dataset to identify synergistic small-molecule pairs active against *E*. *coli*

We first demonstrate that our method for predicting synergistic interactions between small molecules, O2M, can be successfully applied to organisms from different kingdoms. We initially developed O2M for the fungal pathogen *C*. *neoformans*, but here we apply O2M to *E*. *coli* [28] with comparable success. In this section, we describe the initial analysis of the published *E*. *coli* chemical-genetics dataset [28]. In the next section, we show how information from analysis of this dataset allows high-throughput screening for synergistic molecule pairs.

O2M requires a chemical-genetics dataset, generated when a library of knockout mutants is grown in the presence of >100 different chemicals. A quantitative growth score is calculated for each mutant/small-molecule combination. Growth scores can indicate either slower growth (negative values) or faster growth (positive values) compared to wild-type growth scores. The growth scores of all mutants when treated with each small molecule is that small molecule’s “chemical-genetic signature.”

**Fig 1:**
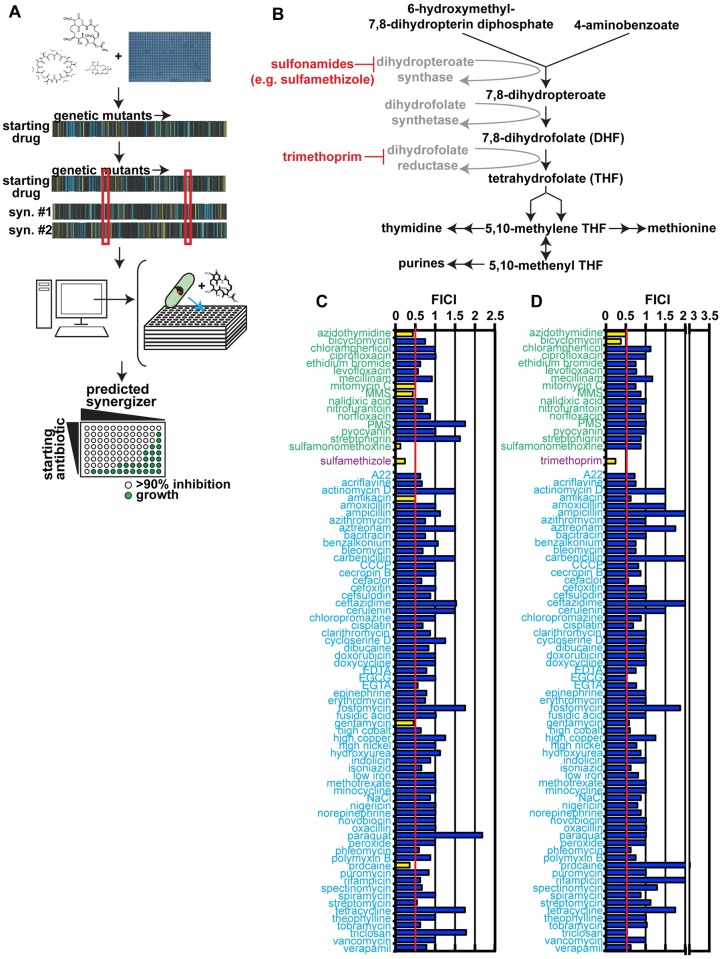
Operon *eck1864-66*Δ mutant serves as a synergy response marker. (A) Outline of the overlap^2^ method (O2M), which we first presented in Brown et al. [18]. O2M requires a chemical-genetic dataset. To generate these datasets, a collection of mutants is grown in the presence of a number of different small molecules. Using colony size as a stand-in for growth, we calculated a quantitative growth score for each combination of mutant + small molecule. From these data, we generate a chemical-genetic signature for each small molecule. This signature includes the score for each mutant in the collection when grown on a particular small molecule. In the heat maps (middle) of “starting drug” versus “genetic mutants”, each vertical line represents a different mutant. A blue line represents small colony size compared to wild-type cells, or a sensitive mutant; a yellow line represents larger colony size, or a resistant mutant. We compare the genetic signatures for starting drugs (e.g., trimethoprim) and known synergistic molecules (e.g., sulfamethizole) computationally. From this analysis, we identify genes whose knockout mutants show the significant growth scores to the starting drug and all its known synergistic partners (outlined by red boxes). These represent the putative synergy prediction mutants. Since our starting drug and its known synergizers induce significant phenotypes from these mutants, we hypothesize that other small molecules that induce significant phenotypes will also synergize with the starting drug. We reanalyze the chemical-genetic dataset to identify these small molecules, then test them in checkerboard analyses. (B) The folate biosynthesis pathway, with trimethoprim and sulfamethizole targets marked. (C) Checkerboard results from trimethoprim + predicted synergistic small molecules (green labels), known synergizer (purple label), and negative control small molecules (blue labels) that are not predicted to synergize with trimethoprim. The fractional inhibitory concentration index (FICI) cutoff for synergy is ≤0.5 (red line), and synergistic FICI values are marked with yellow bars on the graph. Nonsynergistic values are colored blue. Average FICI scores are shown. Individual FICI scores are shown in S2 Table. (D) Predicted synergizers with sulfamethizole. The color scheme is the same as in part C. *P* values were calculated using a Fisher’s exact test. Individual FICI scores are shown in S3 Table.

O2M is based on the rationale that similarities between chemical-genetic signatures of a known synergistic pair contains information that is somehow indicative of synergy—and thus can be used to identify additional synergistic interactions (Fig 1A and [18]). When we compare the chemical-genetic signatures of a pair of small molecules already known to act synergistically, we identified a subset of mutants with similar growth scores. We term this subset of mutants “putative synergy prediction mutants.” We hypothesized that any molecule that elicited the same phenotypes in the same mutants as the known synergistic molecules would also act synergistically with each member of the known synergistic pair.

We analyzed chemical-genetic signatures for the known synergistic antibiotic pair trimethoprim and sulfamethizole from the Nichols et al. *E*. *coli* chemical-genetics dataset [28]. This resource contains quantitative growth scores for over 4,000 *E*. *coli* knockout mutants grown under approximately 300 different conditions (including different types of media and lysogeny broth [LB] medium containing small molecules). We looked for genes whose knockout mutants exhibit a significant (|Z| > 2.5) growth score to both trimethoprim and sulfamethizole (Fig 1B). Genes that are transcribed as a single unit (according to EcoliWiki: http://ecoliwiki.net/colipedia/index.php/Welcome_to_EcoliWiki) were binned together. We identified 4 elements common to both chemical-genetic signatures: *ECK0963-68*, *ECK1082-86*, *ECK1710-13*, *ECK1864-66*, and *ECK3930*. Knockouts of these gene(s) are putative synergy prediction mutants.

We then calculated if each putative synergy prediction mutant successfully identified trimethoprim synergizers. Again, using the Nichols et al. dataset [28], we identified all small molecules that elicit a significant score (|Z| > 2.5) from the 4 putative synergy prediction mutants. We also generated a list of negative control molecules that did not elicit a phenotype from any mutant in any response pattern gene. We performed checkerboard assays, a standard measure of synergistic interactions [29], for each predicted synergizer or negative control combined with trimethoprim or sulfamethizole. All small molecules and their minimum inhibitory concentrations (MICs) are listed in Table 1.

**Table 1:** Minimum inhibitory concentration (90% inhibition) of small molecules used in Fig 1.

Small molecule	MIC (ug/ml)	Category	Biological target
**A22**	1.25	benzodiazipine	cytoskeleton
**acriflavine**	23.4	acridine	DNA
**actinomycin D**	100	actinomycine	transcription
**amikacin**	1.75	aminoglycoside	30S ribosome
**amoxicillin**	1	beta-lactam	cell wall biosynthesis
**ampicillin**	2	beta-lactam	cell wall biosynthesis
**azidothymidine (AZT)**	0.00625	nucleoside analog	DNA
**azithromycin**	10	macrolide	50S ribosome
**aztreonam**	0.01125	monobactam	cell wall biosynthesis
**bacitracin**	7,500	peptide	cell wall biosynthesis
**benzalkonium**	5	cationic surfactant	membrane
**bicyclomycin**	5,000	peptide	transcription termination factor Rho
**bleomycin**	0.05	antineoplastic	DNA
**carbenicillin**	2.5	carboxypenicillin	beta-lactam
**CCCP (Carbonyl cyanide 3-chlorophenylhydrazone)**	31.25	protonophore	oxidative phosphorylation
**cecropin B**	6.25	peptide	membrane
**cefaclor**	6.25	cephalosporin	peptidoglycan
**cefoxitin**	1.4	cephalosporin	peptidoglycan
**cefsulodin**	31.25	cephalosporin	peptidoglycan
**ceftazidime**	0.024	cephalosporin	peptidoglycan
**cerulenin**	62.5	antifungal	fatty acid biosynthesis
**chloramphenicol**	2.5	amphenicols	50S ribosome
**chlorpromazine**	200	antiemetic	MDR transporters
**ciprofloxacin**	0.006	quinolone	DNA gyrase
**cisplatin**	8	antineoplastic	DNA
**clarythromycin**	23	macrolide	50S ribosome
**cobalt stress—CoCl2**	250	metal stress	tRNA methylthio-transferase, aconitase, and ferrichrome reductase
**copper stress—CuSO4**	500	metal stress	oxidative stress
**cycloserine D**	18.75	amino acid derivative	cell wall biosynthesis
**dibucaine**	937.5	anesthetic	membrane
**doxorubucin**	250	anthracycline	topisomerase
**doxycycline**	0.8	tetracycline	30S ribosome
**EDTA**	1.6	chelator	metal metabolism
**EGTA**	100 uM	chelator	metal metabolism
**epigallocatechin gallate (EGCG)**	938	catechin	membrane integrity
**epinephrine**	500	hormone	alpha- and beta-adrenergic receptors (unknown in bacteria)
**erythromycin**	62.5	aminoglycoside	50S ribosome
**ethanol**	100	alcohol	membrane/protein folding
**ethidium bromide**	200	intercaltor	DNA
**fosfomycin**	50	phosphonic antibiotic	cell wall biosynthesis
**fusidic acid**	500	bacteriostat	protein synthesis
**gentamicin**	0.625	aminoglycoside	30S ribosome
**hydroxyurea**	2,500	antineoplastic	DNA
**indolicin**	25	peptide	lipopolysaccharide, membrane
**iron starvation-FeSO(BPS)**	313	iron chelator	iron metabolism
**isoniazid**	1,250	pyridinecarboxylic acid	mycolic acid biosynthesis
**levofloxacin**	0.02	quinolone	DNA gyrase
**mecillinam**	0.3	penicillin	cell wall biosynthesis
**methotrexate**	1,000	folate analog	dihydrofolate reductase
**minocycline**	0.4	tetracycline	ribosome
**mitomycin C**	1	alkylating agent	DNA
**MMS**	25%	alkylating agent	DNA
**NaCl**	1 M	salt	osmotic balance
**nalidixic acid**	1.5	quinolone	DNA gyrase, topoisomerase
**nickel stress-NiCl2**	219	oxidative stress	metal homeostasis
**nigericin**	1,000	ionophore	ion gradients
**nitrofurnatoin**	1.6	hydantoin	ribosome, many macromolecules
**norepinephrine**	26,250	hormone	alpha and beta-adrenergic receptors (unknown in bacteria)
**norfloxacin**	0.025	quinolone	DNA gyrase
**novobiocin**	125	aminocoumarin	DNA gyrase
**oxacillin**	500	penicillin	cell wall biosynthesis
**paraquat dichloride**	15.6	viologen	oxidative stress
**peroxide**	0.01	reactive oxygen species	oxidative stress
**phenazine methosulfate (PMS)**	41	phenothiazine	transport
**phleomycin**	4.1	glycopeptide	DNA
**polymyxin B**	7.1	polymyxin	lipopolysaccharide, membrane
**procaine**	20,000	local anesthetic	unknown in bacteria
**propidium iodine**	500	intercalating agent	DNA
**puromycin**	88	aminonucleoside	protein translation
**pyocyanin**	62.5	toxin	oxidative stress response
**rifampicin**	6.25	antimycobacterial	RNA polymerase
**spectinomycin**	25	aminocyclitol	30S ribosome
**spiramycin**	88.4	macrolide	50S ribosome
**streptomycin**	0.8	aminoglycoside	30S ribosome
**streptonigrin**	12.4	antineoplastic	nucleic acid synthesis
**sulfamethizole**	2	sulfonamide	folic acid biosynthesis
**sulfamonomethoxine**	2.5	sulfonamide	folic acid biosynthesis
**tetracycline**	0.75	tetracycline	30S ribosome
**theophylline**	1,500	methylxanthine	phosphodiesterase, adenosine receptor (unknown in bacteria)
**tobramycin**	0.4	aminoglycoside	30S ribosome
**triclosan**	0.8	biocide	fatty acid biosynthesis, many targets
**trimethoprim**	1	sulfonamide	folic acid biosynthesis
**vancomycin**	62.5	glycopeptide	cell wall biosynthesis
**verapamil**	8,000	calcium channel blocker	efflux pumps

All values are against *E*. *coli* K12.

AZT, azidothymidine; BPS, iron starvation-FeSO; CCCP, Carbonyl cyanide 3-chlorophenylhydrazone; EGCG, epigallocatechin gallate; MDR, multidrug-resistant; MIC, minimum inhibitory concentration; MMS, methyl methanesulfonate; PMS, phenazine methosulfate

A synergistic interaction is defined as at least a 4-fold decrease in the MIC of each drug in the pair, producing a fractional inhibitory concentration index (FICI) of ≤0.5 [30]. We found that small molecules that inhibit growth of putative synergy prediction mutant *eck1864-66*Δ operon are enriched for synergistic interactions with both trimethoprim (*p* < 0.03, Fisher’s exact test) and sulfamethizole (*p* < 0.05, Fisher’s exact test) relative to a randomly generated negative control small-molecule set (Fig 1C & 1D). For example, 25% of predicted synergizers acted synergistically with trimethoprim, compared to 4% of the negative control set. None of the other synergistic response pattern operons identified synergistic interactions with trimethoprim or sulfamethizole at a higher rate than chance (S1 Fig). In total, we identified 5 new synergistic interactions from analyzing the small molecules used in generation of the *E*. *coli* chemical-genetics dataset [28]. Azidothymidine (AZT) acted synergistically with both trimethoprim and sulfamethizole. Three molecules synergize only with trimethoprim and 1 only with sulfamethizole. Since trimethoprim interacted with more molecules, we prioritize it in subsequent experiments.

The genes in the *ECK1864-66* operon are involved in DNA synthesis, modification, and repair, which might explain why this operon, and not our other putative synergy prediction mutants, predicted synergy with trimethoprim. *ECK1864* encodes an endonuclease that resolves Holliday junctions [31,32]. *ECK1865* gene product does not have a known molecular function, but the mutant is sensitive to ionizing radiation [33]. *ECK1866* encodes dihydroneopterin triphosphate pyrophosphohydrolase, an enzyme involved in the early stages of folate biosynthesis [34]. In contrast, the other putative synergy prediction genes, whose mutants did not enrich for trimethoprim synergizers, encoded gene products that did not function in pathways related to DNA synthesis, repair, or folate biosynthesis [35,36].

Notably, these experiments identified new synergistic partners for trimethoprim and sulfamethizole that are not currently used as antibiotics but are approved for human use in other indications. The antiviral drug AZT is promising because of its potent interaction with both trimethoprim and sulfamethizole. AZT has previously reported antibacterial activity but has not been shown to have any synergistic interactions with antibiotics [37–39]. AZT and several other newly identified synergizers are DNA-damaging agents, thereby suggesting a significantly different mechanism of action than the trimethoprim + sulfamethizole combination.

In addition, we performed a similar analysis on vancomycin, which acts synergistically with cephalosporins [40,41]. When we tested the cephalosporins used in the Nichols et al. dataset with vancomycin for synergistic interactions, cefaclor acted synergistically with vancomycin in a checkerboard assay (S2 Fig). We found only 1 gene/operon, *ECK3247-48*, with a mutant that exhibited a significant growth score (|Z| > 2.5) when grown in the presence of vancomycin and cefaclor. We then identified all small molecules from the Nichols et al. dataset [28] that induced a significant phenotype (|Z| > 2.5) from *eck3247*Δ or *eck3248*Δ cells, predicting that these molecules would synergize with vancomycin. When we tested these in checkerboard assays for synergy (S2 Fig), we identified 3 molecules that synergized with vancomycin: chelators EDTA and EGTA and aminocoumarin antibiotic novobiocin. Ion availability is known to effect pathogenicity [42] and vancomycin efficacy [43], but we cannot find previous reports of an interaction between vancomycin and novobiocin, which inhibits DNA gyrase activity [44]. These data demonstrate that O2M identifies synergistic interactions for multiple antibiotics.

### Synergy prediction mutants for trimethoprim allow easy, high-throughput screening for synergistic drug interactions

**Fig 2:**
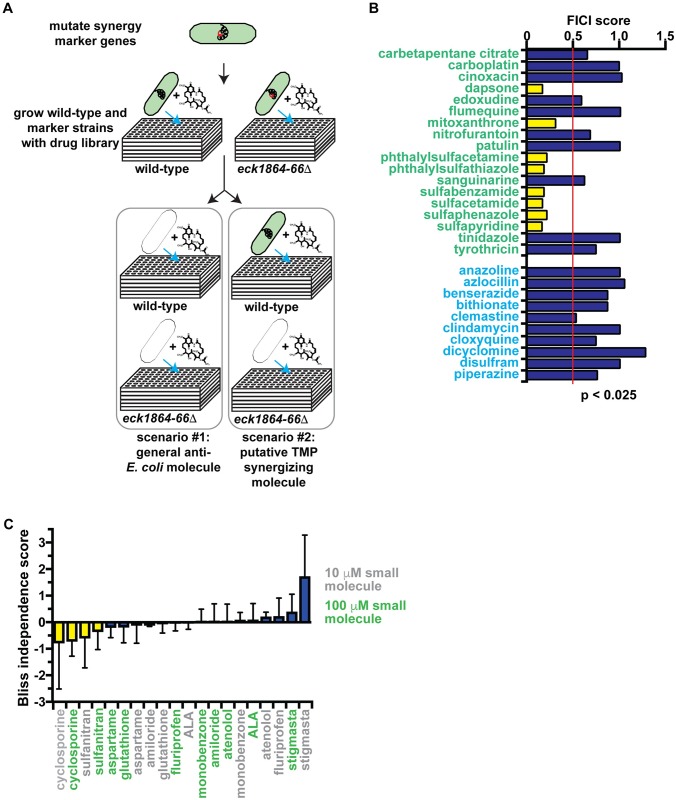
High-throughput screen with synergy response markers. (A) Screen format to identify molecules that synergize with trimethoprim (TMP). (B) Fractional inhibitory concentration index (FICI) of screen hits. Small molecules predicted to synergize with trimethoprim are labeled green. Negative control small molecules, which were part of the Microsource Spectrum collection but not predicted to synergize with trimethoprim, are labeled with blue text. Synergistic FICI values (≤0.5) are marked with yellow bars, and nonsynergistic FICI values are marked with blue bars. Data from this graph are shown in S4 Table. (C) Bliss independence scores for predicted trimethoprim synergizers that do not inhibit *E*. *coli* growth and thus cannot be tested in checkerboard assays. Small molecules were tested at either 10 μM (grey labels) or 100 μM (green labels) in combination with trimethoprim. Small molecules are considered synergistic if they exhibit a negative score at both concentrations (yellow bars). Bars representing data for nonsynergistic small molecules are colored with blue. Data from this graph are shown in S5 Table.

We exploited our newly identified synergy prediction mutant (*eck1864-66*Δ) to perform one of the first high-throughput screens for synergistic pairs (Fig 2A). Our rationale was that because synergy prediction mutants exhibited the same phenotypic response to molecules known to act synergistically, this limited set of knockout mutants could be used to rapidly screen additional small molecules to identify those that are likely to be synergistic with the starting molecule.

Our assay is extremely simple and identifies synergistic pairs without performing multidrug assays. Instead, synergy prediction mutants functionally substitute for 1 of the antibiotics. We screened the Microsource Spectrum Collection, a small-molecule library of 2,000 compounds that is enriched for Food and Drug Administration (FDA)-approved drugs. We grew wild-type and *eck1864-66*Δ mutant cells in the presence of each small molecule, then identified small molecules that inhibit growth of the synergy response marker strain but not wild-type cells after 18 hours of growth (Z score < −2.5). We identified 28 of these putative trimethoprim-synergizing molecules (Table 2).

**Table 2:** Predicted trimethoprim synergizers from Microsource Spectrum collection screen.

Small molecule	MIC (ug/ml)	Class	Biological target	FDA approved
4-Aminophenyl sulfone (dapsone)	50	sulfone	folate biosynthesis	yes
amiloride HCl	no inhibition	potassium-sparing diuretic	sodium channels	yes
aminolevulinic acid HCl	no inhibition	photosensitizing agent	porphyrin biosynthesis (activator)	imaging agent
aspartame	no inhibition	peptide	artificial sweetener	food product
atenolol	no inhibition	beta blocker	beta1 receptor (mammals)	yes
carbetapentane citrate	no inhibition	antitussive	muscarine receptors	yes
carboplatin	1,875	antineoplastic	DNA	yes
cinoxacin	2.5	quinolone	DNA gyrase	discontinued
cyclosporine	no inhibition	immunosuppressamt	calcineurin	yes
edoxudine	3.9	nucleoside analog	DNA	yes
flumequine	0.625	fluoroquinolone	DNA gyrase	discontinued
flurbiprofen	no inhibition	NSAID	cyclooxygenase	yes
glutathione	no inhibition	N/A	reactive oxygen species	no
karanjin	no inhibition	N/A	nitrification	no
mitoxanthrone HCl	39	antineoplastic	DNA	yes
monobenzone	no inhibition	quinone	melanization	topical
nitrofurantoin	1.6	nitrofuran	broad	yes
patulin	2.5	N/A	potassium uptake	no
phthalylsulfactamide	1,250	sulfonamide	folate biosynthesis	yes
phthalylsulfathiazole	50	sulfonamide	folate biosynthesis	yes
sanguinarine chloride	10	N/A	apoptosis	no
stigmasta-4,22-dien-3-one	no inhibition	N/A	unknown	no
sulfabenzamide	7.8	sulfonamide	folate biosynthesis	discontinued
sulfanitran	no inhibition	sulfonamide	multidrug resistance transporter	yes
sulfaphenazole	3.1	sulfonamide	folate biosynthesis	yes
tinidazole	1,250	nitroimidazole	DNA	yes
tyrothricin	47	polypeptide	cytoplasmic membrane	yes

Verification data is shown in Fig 2.

FDA, Food and Drug Administration; MIC, minimum inhibitory concentration, N/A, not applicable; NSAID, nonsteroidal anti-inflammatory drug

We verified the synergistic interactions between trimethoprim and our screen hits using 2 different methods: checkerboard assays and Bliss Independence. Checkerboards are preferable but require that both small molecules inhibit microbial growth on their own. Of the 18 screen hits that met this criterion, 8 were verified to act synergistically with trimethoprim (Fig 2B). This 44% enrichment rate is significantly (*p* < 0.05) greater than the 4% frequency of trimethoprim synergizers in a randomly selected set of small molecules (Fig 1). Six of these small molecules (phthalylsulfacetamide, phthalysulfathiazole, sulfabenzamide, sulfacetamide, sulfaphenazole, and sulfapyridine) are sulfonamide antibiotics that inhibit dihydropteroate synthetase, the same target as sulfamethizole. A seventh, dapsone, also inhibits dihydropteroate synthetase but belongs to a different class of drugs [45]. The final verified screen hit, mitoxanthrone, is an antineoplastic DNA-intercalating agent [46] and not a sulfonamide antibiotic.

The remaining 10 screen hits do not inhibit *E*. *coli* growth on their own, so we attempted to verify their synergistic action using the Bliss independence model [30]. Briefly, in a 96-well plate containing growth medium and bacteria, we created a gradient of trimethoprim, then added each small molecule of interest at both 10 μM and 100 μM concentrations. Synergistic small molecules enhance growth inhibition by trimethoprim at both concentrations versus trimethoprim alone. We found that 2 of 10 small molecules exhibit synergy at both concentrations (Fig 2C). Sulfanitran is a sulfonamide antibiotic. Cyclosporine is a cyclic peptide that inhibits calcineurin [47] but is not known to have a bacterial target.

The new synergistic molecules found from O2M analysis and the high-throughput screen fall into 2 main groups. First, the sulfonamide antibiotics almost certainly act by the same mechanism as trimethoprim + sulfamethizole, so strains resistant to the combination would likely also be resistant to these new pairs. The second group consists of several DNA damaging agents (AZT, mitomycin C, mitaxanthrone). This result suggests a second molecular mechanism underlying synergy. Therefore, we focus on this second group in subsequent experiments.

### Synergistic drug pairs are active against multidrug-resistant clinical *E*. *coli* and *K*. *pneumoniae* isolates

Our most promising new synergistic interaction is between trimethoprim or sulfamethizole and AZT. The first anti-HIV drug [48], AZT, was investigated as a chemotherapeutic before the discovery of its antiretroviral activity [49]. AZT’s MIC against multidrug-resistant (MDR) *E*. *coli* and *K*. *pneumoniae* is in the nanogram per milliliter range (Table 3), suggesting that it could be a powerful antibiotic. AZT causes premature chain termination during bacterial DNA replication [38,39,50], induces the SOS response [38,39], and moderately increases mutation rates [51].

**Table 3:** Minimum inhibitory concentration (90% inhibition) of trimethoprim, sulfamethizole, AZT, and floxuridine against clinical isolates.

strain	species	Minimum inhibitory concentration (μg/ml)				
		trimethoprim	sulfamethizole	AZT	hydroxyurea	floxuridine
blood isolate #1	*E*. *coli*	0.5	62.5	1.0	1,250	0.02
blood isolate #2	*E*. *coli*	1.0	62.5	0.5	625	0.04
blood isolate #3	*E*. *coli*	0.5	1.25	0.08	312.5	0.005
blood isolate #4	*E*. *coli*	2.0	5,000	1.1	625	0.04
blood isolate #5	*E*. *coli*	1,025	205	0.25	625	0.04
blood isolate #6	*E*. *coli*	1.0	31.25	2.1	625	0.04
blood isolate #7	*E*. *coli*	3.0	5,000	2.1	625	0.03
blood isolate #8	*E*. *coli*	1,025	205	0.11	312.5	0.02
blood isolate #9	*E*. *coli*	250	6.25	0.5	625	0.04
UTI isolate #1	*E*. *coli*	1,025	205	0.5	625	0.015
UTI isolate #2	*E*. *coli*	1,025	205	0.5	312.5	0.03
UTI isolate #3	*E*. *coli*	1,025	205	0.03	312.5	0.01
clinical isolate #1	*K*. *pneuomoniae*	3,000	6.25	0.13	2,500	0.01
clinical isolate #2	*K*. *pneuomoniae*	3,000	5,000	0.04	1,250	0.008
clinical isolate #3	*K*. *pneuomoniae*	3,000	5,000	0.27	1,250	0.04
clinical isolate #4	*K*. *pneuomoniae*	3,000	5,000	0.27	1,250	0.04
clinical isolate #5	*K*. *pneuomoniae*	3,000	2,500	0.53	1,250	0.08

AZT, azidothymidine; UTI, urinary tract infection

**Fig 3:**
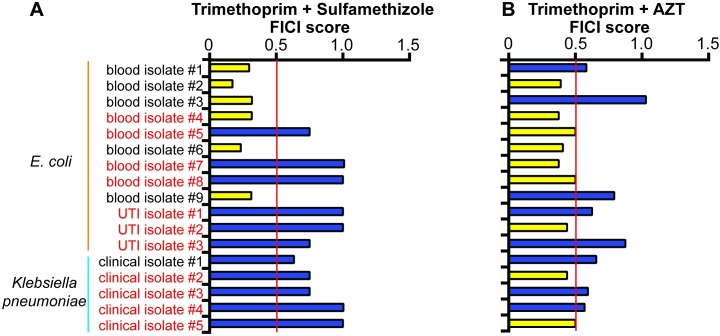
Trimethoprim and azidothymidine (AZT) act synergistically in clinical strains that do not respond to trimethoprim and sulfamethizole. (A) Trimethoprim + sulfamethizole. (B) Trimethoprim + AZT. Synergistic fractional inhibitory concentration index (FICI) values (≤0.5) are marked with yellow bars, and nonsynergistic (FICI > 0.5) FICI values are marked with blue bars. Trimethoprim/sulfamethizole-resistant isolates are labeled with red text and sensitive isolates are labeled with black text. Average FICI scores are shown in the graph. Individual FICI scores are shown in S6 Table.

We tested whether AZT acts synergistically with trimethoprim in 12 MDR clinical isolates of *E*. *coli* and 5 MDR *K*. *pneumoniae* isolates. Seven of *the E*. *coli* isolates and 4 *K*. *pneumoniae* isolates are resistant to the trimethoprim/sulfamethizole combination. In the vast majority of trimethoprim/sulfamethizole-resistant isolates, the classic combination of trimethoprim and sulfamethizole no longer acted synergistically (FICI ≤ 0.5) (Fig 3A). One possible reason behind this is that trimethoprim and sulfamethiole targets are in the same pathway [52], so resistance to 1 drug could confer some resistance to the other and block the synergistic interaction. In contrast, trimethoprim and AZT acted synergistically against 5 *E*. *coli* and 2 *K*. *pneumoniae* trimethoprim/sulfamethizole-resistant isolates. Our new synergistic pair thus acts against multiple species of trimethoprim/sulfamethizole-resistant bacteria.

### The synergistic interaction between trimethoprim and AZT activates a stress pathway that the single agent drugs do not

The mechanisms underlying synergistic interactions are poorly explored, with the exception of trimethoprim + sulfamethizole (or other sulfamonamides). Both molecules are inhibitors of folate biosynthesis, so these drugs were historically thought to synergize due to simultaneous inhibition of 2 enzymes in the folate biosynthesis pathway [52]. The Nichols et al. chemical-genetic analysis suggests that trimethoprim and sulfonamides differentially impact the steps between tetrahydrofolate and 5,10-methylene tetrahydrofolate production (Fig 1B) [28]. Regardless, since DNA-damaging agents such as AZT do not inhibit folate biosynthesis, they likely act through a second mechanism of synergy.

AZT alone induces the SOS response [38,39], so we hypothesized that the synergistic pairing with trimethoprim could amplify each molecule’s individual activity. We performed checkerboard assays on K12 *E*. *coli* carrying a green fluorescent protein (GFP) reporter plasmid under control of the SOS-induced *sulA* promoter. We selected the *sulA* promoter because it is induced late in the SOS response, indicating a robust SOS response and cell growth arrest [53].

**Fig 4:**
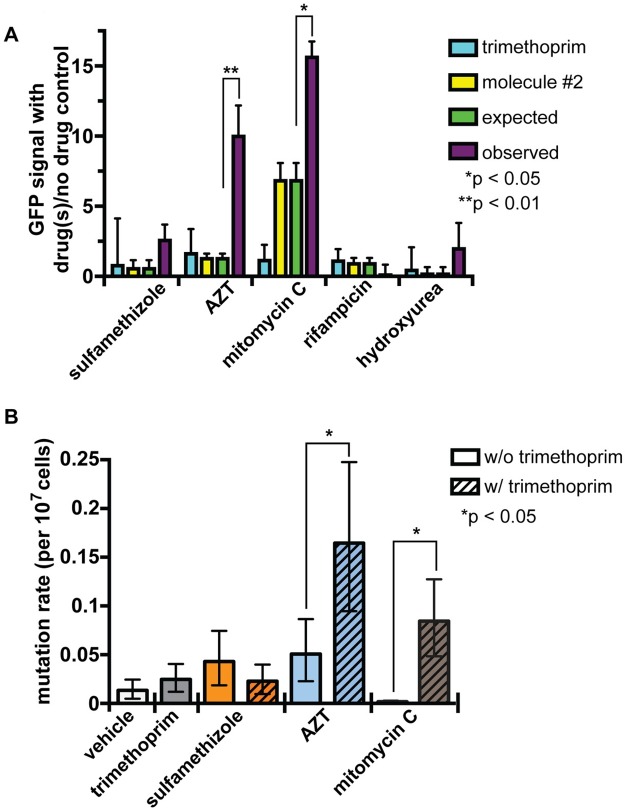
The SOS response is induced by trimethoprim and azidothymidine (AZT). (A) SOS response measured by a green fluorescent protein (GFP) reporter gene under control of the *sulA* reporter. Small molecules were added at 50% minimum inhibitory concentration (MIC). Expected *sulA* induction (green) is either the induction by the second molecule or, if the *sulA* reporter is repressed by the second molecule, no induction or repression. Trimethoprim does not induce the *sulA* reporter, so it is considered to not have any contribution to the expected value, and we do not simply sum the induction of trimethoprim + molecule #2. The observed *sulA* induction (purple) is significantly higher than expected in the trimethoprim and AZT combination but not nonsynergistic combinations, such as trimethoprim + hydroxyurea or trimethoprim + rifampicin. Since the trimethoprim + sulfamethizole combination does not induce *sulA*, the molecular mechanisms underlying trimethoprim + sulfamethizole synergy likely differ from trimethoprim + AZT synergy. Significance was calculated using a Mann-Whitney test. Error bars represent the standard deviation. In all cases when we observed a significant difference between expected and observed, we also found a significant difference between induction by molecule #2 and induction in the combination. The data for these graphs are in S7 Table. (B) Fluctuation assay measures the mutation rate following small-molecule treatment. Small molecules alone (solid colors) do not significantly increase mutation rate. Trimethoprim combined (striped bars) with synergistic partners AZT or mitomycin C increases mutation rate. *P* values were calculated using Fisher’s exact test. Error bars represent the 95% confidence interval. The data for these graphs are in S8 Table.

Neither trimethoprim nor sulfamethizole alone induces the *sulA* reporter compared to a no-drug control (Fig 4A). AZT alone induced the *sulA* promoter modestly but reproducibly (1.6-fold relative to the control). These results predict that the combination of AZT and trimethoprim would show a 3-fold induction. Instead, we see a 9-fold induction (*p* < 0.01; Mann-Whitney test). We observed the same trend for mitomycin C, a DNA crosslinking agent [54] that also synergizes with trimethoprim (Fig 1).

We also tested 2 additional molecules as controls. The RNA polymerase inhibitor rifampicin, which blocks the SOS response [55] and does not synergize with trimethoprim (Fig 2), exhibited a lower SOS response in combination with trimethoprim than alone (*p* < 0.005; Mann-Whitney test). Similarly, the DNA-damaging agent hydroxyurea (HU) [56], which does not synergize with trimethoprim (Fig 2), also does not induce the *sulA* promoter alone or in combination with trimethoprim.

Since the SOS response induces error-prone DNA repair, we hypothesized that the combination of trimethoprim and AZT increases mutation burden beyond that caused by each small molecule alone. To test this hypothesis, we performed a fluctuation assay to measure the mutation rate [57]. We grew cells in subinhibitory concentrations of each small molecule alone or in combination, then plated cells to LB + 15 μg/ml nalidixic acid, which selects for mutations in the topoisomerase gene [58,59]. We calculated mutation rate from the number of resistant colonies within the total population [60].

The trimethoprim synergizers AZT and mitomycin C both increase mutation rate by at least 3-fold in combination with trimethoprim but not alone, even at the subinhibitory concentrations tested (1/8 of MIC) (Fig 4B). Sulfamethizole alone or in combination with trimethoprim does not increase mutation rates. These data suggest that the amplification of DNA damage is an important step in the synergistic interaction between trimethoprim and AZT (or mitomycin C), while trimethoprim and sulfamethizole interact through a different mechanism.

### Genetic or chemical reduction of deoxynucleotide pools sensitizes *E*. *coli* cells to AZT

Our data support the model that DNA damage accumulates in cells treated with trimethoprim + AZT. AZT’s connection to DNA damage is clear from its known mechanism of action [39]. However, trimethoprim’s connection is indirect. Folate is necessary for the biosynthesis of purines, thymidine [61], and methionine [62], and treatment with trimethoprim disrupts nucleotide homeostasis [61]. We hypothesized that such reduced nucleotide availability amplifies the phenotypic consequences of premature chain termination caused by AZT. Should the synergistic interaction between trimethoprim and AZT be due to the simultaneous inhibition of chain termination and depleted nucleotide pools, then we would expect additional, unrelated small molecules that cause similar effects to also interact synergistically. Furthermore, genetic mutations that deplete nucleotide pools would result in increased sensitivity to AZT, and other chain-terminating agents would cause increased sensitivity to trimethoprim.

**Fig 5:**
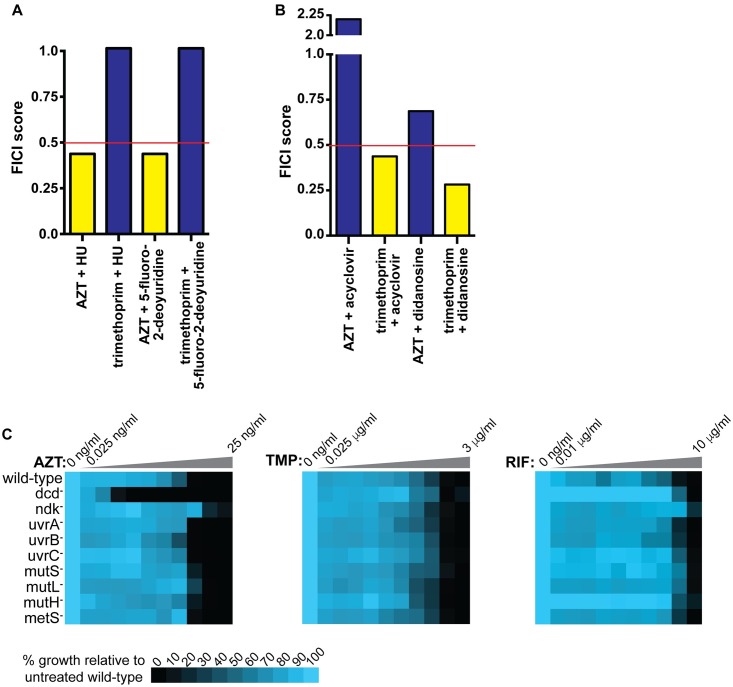
The combination of disrupted nucleotide balance and premature DNA chain termination act synergistically to inhibit *E*. *coli* growth. Bars for nonsynergistic combinations (fractional inhibitory concentration index [FICI] > 0.5) are colored blue, and bars for synergistic combinations (FICI ≤ 0.5) are colored yellow. Checkerboard assays for (A) nucleotide homeostasis disruptors + azidothymidine (AZT) or trimethoprim and (B) Nucleoside analogs + AZT or trimethoprim show synergy between nucleotide homeostasis inhibitors and AZT or nucleoside analogs and trimethoprim but not the reverse. (C) Growth of wild-type and mutant cells on AZT, trimethoprim (TMP) or rifampicin (RIF). These data are the average of 3 replicates. The data for parts A and B are in S9 Table. The data for part C are in S10 Table.

First, we tested the proposed nucleotide homeostasis/DNA chain termination interaction chemically. We performed a checkerboard assay with AZT and HU or floxuridine, 2 FDA-approved drugs that alter deoxynucleotide triphosphate (dNTP) balance [63]. Both HU and floxuridine acted synergistically with AZT but not trimethoprim (Fig 5A). We then tested trimethoprim in combination with nucleoside analogs other than AZT. As expected, nucleoside analogs that inhibit *E*. *coli* growth interact synergistically with trimethoprim but not AZT (Fig 5B).

Second, this interaction between nucleotide pools and AZT also occurs genetically. The *E*. *coli* gene deoxycytidine deaminase (*dcd*) encodes deoxycytidine triphosphate (dCTP) deaminase [64]. *dcd* deletion mutants exhibit depleted deoxythymidine triphosphate (dTTP) pools and elevated dCTP pools when grown in minimal medium [64–66]. *dcd* deletion mutant cells exhibit a 32-fold increase in AZT sensitivity compared to wild-type cells but no change in trimethoprim or rifampicin sensitivity (Fig 5C). By contrast, mutants in nucleoside diphosphate kinase (ndk) [67] exhibit elevated dCTP and dTTP pools but lower deoxyadenosine triphosphate (dATP) pools [66,68]. *ndk* deletion mutant cells exhibit a 4-fold decrease in AZT sensitivity (increased resistance) compared to wild-type cells. These results suggest that adequate dTTP is necessary for surviving AZT exposure. Therefore, we conclude that simultaneous disruption of nucleotide homeostasis and DNA replication increase *E*. *coli* growth inhibition.

We next tested if DNA repair mutants in general are hypersensitive to AZT or if the hypersensitivity is specific to disrupted nucleotide homeostasis. We grew cells deficient in mismatch repair (*mutL*, *mutS*, or *mutH* deletion mutants) or nucleotide excision repair (*uvrA*, *uvrB*, or *uvrC* deletion mutants) in AZT, trimethoprim, and rifampicin and did not observe increased sensitivity to any of these (Fig 5C). Finally, to make sure that the interaction between AZT and trimethoprim was not due to methionine depletion, we tested a deletion mutant in the methionine synthase gene *metH* [62]. *metH* mutant cells exhibited wild-type levels sensitivities to AZT, trimethoprim, and rifampicin (Fig 5C). Therefore, we conclude that disruption of nucleotide homeostasis (by multiple possible mechanisms) hypersensitizes bacterial cells to DNA damage caused by AZT. By substituting HU or floxuridine for trimethoprim, we demonstrated that we can rationally design additional synergistic pairs once the molecular mechanism underlying an interaction is understood.

### Small molecules that disrupt nucleotide homeostasis act synergistically with AZT against clinical *E*. *coli* and *K*. *pneumoniae* isolates

**Fig 6:**
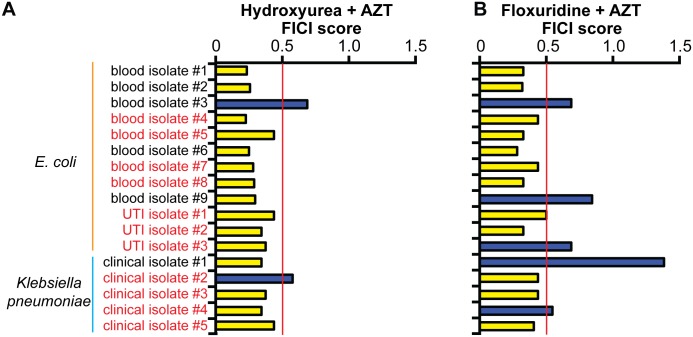
Trimethoprim replacement molecules act synergistically with azidothymidine (AZT) against clinical isolates. Synergistic fractional inhibitory concentration index (FICI) values (≤0.5) are marked with yellow bars, and nonsynergistic (FICI > 0.5) FICI values are marked with blue bars. Trimethoprim/sulfamethizole-resistant isolates are labeled with red text, and sensitive isolates are labeled with black text. (A) Hydroxyurea (HU) + AZT or (B) floxuridine + AZT. Individual FICI scores for this graph are listed in S11 Table. Example checkerboard data are shown in S3 Fig.

Finally, we tested our new, rationally designed synergistic pairs against our collection of clinical *E*. *coli* and *K*. *pneumoniae* strains. Substituting either HU (Fig 6A) or floxuridine (Fig 6B) for trimethoprim, we found that either molecule combined with AZT inhibited growth of trimethoprim/sulfamethizole-resistant isolates. HU + AZT acted synergistically in 15 of 17 clinical isolates, including all but 1 of the trimethoprim/sulfamethizole-resistant isolates (*n* = 11). Floxuridine + AZT also acted synergistically in most clinical isolates (12 of 17), including all but 2 trimethoprim/sulfamethizole-resistant isolates. The MICs of floxuridine and AZT are sub-μg/ml for most MDR clinical isolates, whereas MICs for trimethoprim or sulfamethizole were up to 5,000 μg/ml in vitro (Table 3). Rationally designed synergistic pairs therefore bypassed resistance in clinical isolates.

### The new floxuridine-AZT combination shows markedly improved efficacy in a zebrafish infection model

**Fig 7:**
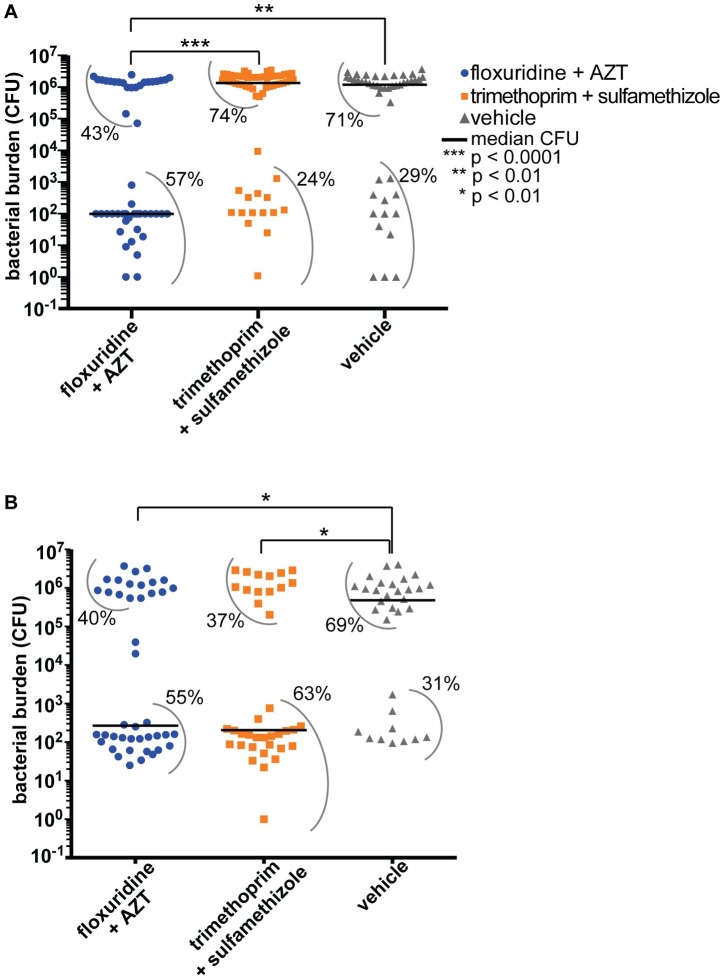
The rationally designed synergistic combination of floxuridine + azidothymidine (AZT) improves treatment of infected zebrafish embryos. (A) We injected a trimethoprim/sulfamethizole-resistant *E*. *coli* strain (blood isolate #8, or BEC8) into zebrafish embryos, then treated them with drugs starting at 3 hours postinoculation (hpi). At 24 hpi, we euthanized embryos and determined bacterial burden (colony-forming units [CFU]) in whole fish. Each symbol (blue circles for floxuridine + AZT, orange squares for trimethoprim [TMP] + sulfamethizole [SFZ], and grey triangles for vehicle control) represents a single fish. *N* ≥ 30 each condition. Data from 3 separate experiments are shown. Inoculum levels are shown in S4 Fig. Black lines represent median bacterial burden for each condition. *P* value was calculated using a Mann-Whitney test. Grey arcs show the percent of individual embryos within each population group. For example, 57% of the floxuridine + AZT-treated group has a bacterial burden between 1 CFU and 10^3^ CFU. (B) Zebrafish embryos infected with *E*. *coli* strain F11, which is sensitive to trimethoprim/sulfamethizole. The color scheme and symbols are the same as in part A. Bacterial burden for each individual embryo are listed in S12 Table. Fractional inhibitory concentration index (FICI) information for strain F11 is in S13 Table.

We then tested the floxuridine + AZT combination in a zebrafish infection model. Efficacy of synergistic drug pairs has historically been difficult to evaluate in vertebrate systems, and many prior studies use the moth larvae *Galleria mellonella* or perform only in vitro tests [19,20,69,70]. Zebrafish offer several advantages: they have a mammalian-like innate immune system [71], a long history as microbial infection models [71–75], and are a good platform for assessing drug toxicity [76,77]. We injected approximately 2,500 colony-forming units (CFU) of trimethoprim-resistant *E*. *coli* into the pericardial cavity of zebrafish embryos [75,78], incubated for 3 hours, then treated with either the original drug combination (trimethoprim + sulfamethizole) or our rationally designed combination (floxuridine + AZT). We analyzed bacterial burden at 24 hours postinoculation (hpi) at dosages analogous to human dosage (see Materials and methods and Table 4) [79,80]. The new combination was indeed successful—it resulted in a 10,000-fold reduction in median bacterial burden in infected zebrafish embryos treated with floxuridine + AZT compared to infected embryos treated with trimethoprim + sulfamethizole (Fig 7A). When we infected embryos with a trimethoprim/sulfamethizole-sensitive *E*. *coli* strain, floxuridine + AZT treatment and trimethoprim + sulfamethizole treatment were equally effective. (Fig 7B).

**Table 4:** Drug doses in zebrafish infection experiment.

Drug	Human dose			Zebrafish dose		
	*oral administration*	*IV administration*	*condition*	*yolk injection*	*estimated dose (embryo approximately 300* μ*g)*	*treatment concentration in water*
AZT	600 mg/day	5–6 mg/kg/day	HIV	0.006 pg	2 x 10^−5^ mg/kg	6 ng/ml
Floxuridine	–	0.1–0.4 mg/kg/day	adenocarcinoma	0.048 pg	1.6 x 10^−4^ mg/kg	48 ng/ml
Trimethoprim	320 mg/day	1.6–2 mg/kg/day	UTI, bronchitis, polynephritis	0.120 pg	4 x 10^−4^ mg/kg	120 ng/ml
Sulfamethizole	1,600 mg/day	8–10 mg/kg/day	UTI, bronchitis, polynephritis	0.6 pg	2 x 10^−3^ mg/kg	600 ng/ml

Human dosages and routes of administration are shown on the left (data from drugs.com). When possible, we used the human oral dose of the drug in the zebrafish water, although trimethoprim and sulfamethizole were slightly toxic to embryos at these concentrations, so we lowered the doses to those shown. Floxuridine is administered by IV, but since the oral dose of the other drugs are 100 to 200 times the IV dose, we used a dose within that range (120 times the IV dose).

AZT, azidothymidine; UTI, urinary tract infection

We also analyzed MICs in the presence of human serum, as a substantial increase in MIC in the presence of serum would indicate that our small molecules of interest are binding to serum proteins and are not bioavailable [81]. We tested MICs of floxuridine, AZT, trimethoprim, and sulfamethizole with and without 20% human serum and found only minor changes in MIC (S14 Table). The dosages we use in zebrafish are well under the human dosage (Table 4), suggesting that it would be possible to obtain the necessary drug concentrations in humans.

## Discussion

### High-throughput identification of synergistic small-molecule pairs

Synergistic small molecules are of considerable clinical interest, but systematic identification has been challenging. This study describes 4 significant advances to such systematic identification. First, we demonstrate that O2M is generally applicable beyond the fungal pathogen for which it was originally developed [18]. Second, we and others show that previously published chemical-genetic datasets [18,22–26,28,82] can be successfully used as the raw input for O2M, significantly decreasing the upfront investment required. Third, O2M identifies knockout mutants that can be used as readouts for synergy in highly scalable screening assays for additional synergistic combinations. Finally, understanding the mechanisms that underlie synergistic interactions can facilitate the rational design of further synergistic combinations that bypass antibiotic resistance.

Our results show that a wide variety of synergistic combinations are available if we know how to search for them. We would suggest that these discoveries represent a small fraction of the potential synergistic combinations. In support of this idea, several groups recently published analysis methods that, like our original O2M analysis [18], use chemical-genetics data to predict synergy between antibiotics [19] or antifungals [20,21,70]. Notably, each method identifies different, complementary synergistic pairs, suggesting that current methods are far from identifying all synergistic interactions.

As described here, the particular advantage of O2M is its scalability to screen for synergistic small molecules that are not commonly used as antibiotics. This scalability is critical to keeping the initial screens as broad as possible—since substituting 1 member of a synergistic pair for a second member can change the molecular mechanism underlying the interaction, identification of diverse synergistic pairs offers the greatest potential for further rational design. We screened a well-studied collection of FDA-approved small molecules, identifying 14 novel synergistic combinations with the widely used antibiotic trimethoprim. O2M is also much faster than a pairwise screen of a small-molecule library. By identifying synergy prediction mutants, we can screen 2,000 molecules and verify only those predicted to be synergistic. The 28 predicted synergistic molecules identified in our trimethoprim/sulfamethizole screen took less than a week to validate. Testing of the entire 2,000 small-molecule collection in combination with a single molecule would take months.

### Mechanisms underlying synergistic small-molecule interactions

**Fig 8:**
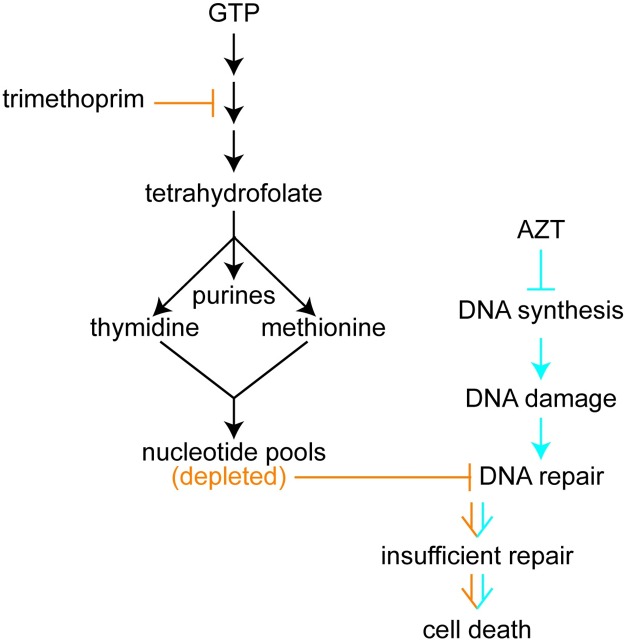
Inhibition of nucleotide homeostasis amplifies the consequences of DNA damage, increasing the toxicity of DNA-damaging agents. Trimethoprim treatment (orange graphics) blocks DNA repair, which is induced by azidothymidine (AZT) treatment (blue graphics). The combined effects result in increased growth inhibition relative to single-agent treatment.

The molecular mechanisms underlying synergistic drug interactions are generally poorly understood [4,28]. There are 3 main hypotheses for why any given pair of small molecules exhibit synergistic interaction: that the pairs (1) act together to cause a third, novel inhibitory activity (“gain-of-function” hypothesis), (2) act in combination by simultaneously inhibiting 2 different functions to increase potency (“two-hit” or “parallel pathway” hypothesis) [83], or (3) 1 drug increases the activity and/or bioavailability of the other (“bioavailability” hypothesis) [84]. Our data demonstrate that the trimethoprim and AZT interaction likely represent a “two-hit” mechanism, acting through the combined induction of DNA damage and blocking DNA repair by disrupting nucleotide homeostasis (Fig 8). This molecular mechanism quite likely differs from that historically thought to underlie the trimethoprim/sulfonamides [52]. Recent data suggest that the trimethoprim + sulfamethizole interaction is not as simple as simultaneous inhibition of 2 folate biosynthesis enzymes [52]. Instead, the hypothesis is that trimethoprim and sulfonamides result in buildup of different secondary metabolites, which differentially impact enzyme activities [28]. However, since the phenotypic consequences of treatment with trimethoprim + sulfamethizole differ from treatment with trimethoprim + AZT, the mechanisms are likely also different. We surmise that both combinations represent “two-hit” synergistic interactions. “Two-hit” synergistic interactions can be predicted from network analysis: genes/pathways whose knockouts exhibit synthetic lethality could be good targets of drug combinations. These analyses also demonstrate the importance of network analysis and high-throughput studies on model organisms [85]: the chemical-genomic dataset we used for our initial O2M analysis was from a nonpathogenic K12 genetic background [28], showing that K12 data are sufficient to elucidate synergistic drug mechanisms.

One potential concern about using trimethoprim + AZT or floxuridine + AZT clinically is that these combinations increase mutation rate. While this is indeed a concern, many antibiotics target DNA replication and other processes that also increase mutation rates. Our measured mutation rate for trimethoprim + AZT, approximately 2 x 10^−8^ mutations per cell, is well within the range of other antibiotics [86]. These vary from 10^−9^ mutations per cell (clarithromycin and amoxicillin) [86] to 10^−6^ mutations per cell [87]. Mutation rates will vary with species and strain [87,88], but trimethoprim + AZT induces mutation rates comparable to those induced by ciprofloxacin [86].

### Towards rational design of synergistic combination therapy

Once we identify biological pathways and processes whose simultaneous inhibition blocks microbial growth, we can then rationally design synergistic drug treatments that bypass antibiotic resistance. Here, we present a proof-of-principle methodology that demonstrates the power of this rational design. Once we identified the molecular mechanism underlying the trimethoprim + AZT interaction, we substituted trimethoprim for another FDA-approved small molecule. This newly designed combination, floxuridine + AZT, achieved the same synergistic interaction with AZT yet bypassed trimethoprim resistance in MDR clinical isolates (Figs 6 and 7). Indeed, floxuridine + AZT was far superior to trimethoprim + sulfamethizole in a vertebrate infection model with trimethoprim/sulfamethizole-resistant *E*. *coli*. That is, we inhibited MDR *E*. *coli* infection with lower doses of structurally unrelated but functionally similar small molecules.

The optimal clinical application of interacting small molecules is currently under debate. Recent work suggested that sequential, rather than simultaneous, application of synergistic small molecules prevents the development of drug resistance [8,89]. Others suggest that antagonistic small-molecule interactions could be beneficial [8,83,90]. Antagonistic interactions occur when 2 molecules in combination decrease each other’s efficacy. One theory is that antagonism decreases the selective advantage of a drug-resistant mutation, and thus evolution of resistance is slower to an antagonistic pair than a synergistic pair [90]. These ideas merit further exploration in clinical and animal models of infection.

In sum, O2M is an important tool for high-throughput identification of synergistic small-molecule pairs and successfully identifies new treatments to combat MDR infections. With the growing antibiotic crisis, treatments that are effective against MDR bacteria need to move rapidly into the clinic. Our method of screening FDA-approved drugs identified candidate treatments that could be deployed with fewer regulatory trials than needed for new drugs. Moreover, our rationally designed treatment, floxuridine + AZT, also uses FDA-approved agents (and AZT is well tolerated for short-term treatments, despite toxicities associated with long-term use at high doses [91]). We hope to spur interest among clinicians to test such designed synergistic combinations against difficult-to-treat MDR infections, when appropriate.

## Materials and methods

### Ethics statement

Animals used in this study were handled in accordance with protocols approved by the University of Utah IACUC committee (protocol 10–02014), which follow guidelines from the Guide for the Care and Use of Laboratory Animals and zfin.org. Zebrafish older than 3 days postfertilization were euthanized by immersion in a chilled water bath followed by mechanical disruption. Zebrafish younger than 3 days, which do not have developed pain sensors, were euthanized by mechanical disruption. Infections took place under tricaine anesthesia.

### *E*. *coli* strains

Unless otherwise stated, experiments were performed on *E*. *coli* K12 strain MG1655. *E*. *coli* blood isolates #1–8 (referred to as BEC1, BEC2, etc.) are described in Barber et al. [78]. Urinary tract infection (UTI) isolates and *K*. *pneumoniae* isolates were obtained from ARUP labs. Strains are listed in S15 Table.

### *E*. *coli* growth and small-molecule assays

All assays were performed in M9 minimal medium (10.5g/L M9 broth [Amresco], 0.2% casamino acids, 0.1M CaCl_2_, 0.4% glucose, 1M MgSO_4_, 0.25% nicotinic acid, 0.33% thiamine in H_2_O) unless otherwise stated. To determine MICs, an M9 culture of MG1655 was growth overnight at 37°C with shaking, then diluted to OD_600_ = 0.002. We then inoculated each well with approximately 1,000 cells (2 μl of culture into 200 μl of medium per well). Plates were incubated at 37°C unless otherwise stated. Small-molecule gradients were diluted in 2-fold dilution series unless otherwise stated. MIC values (Table 1) are calculated following 24 hours incubation at 37°C. MIC values are calculated as >90% growth inhibition unless otherwise stated. MIC values for MDR strains, which grow more rapidly than lab strains, were calculated following 24 hours incubation at 37°C. When calculating MICs in the presence of human serum, we used standard techniques but substituted up to 20% of the media volume with human serum (Sigma).

### O2M analysis

We performed O2M analysis as previously described [18] with the following variations. (Step-by-step instructions are available in the S1 Text section). The Nichols et al. paper calculates a growth score for each mutant + small molecule combination [28]. We then use these growth scores to calculate significance in O2M analysis. We considered any growth score significant if either: (1) growth score for mutant A and small molecule B ≥ average growth score for all mutants when grown on plates containing small molecule B + 2.5*standard deviation of all small molecule B growth scores, or (2) growth score for mutant A and small molecule B ≤ average growth score for all mutants when grown on plates containing small molecule B − 2.5*standard deviation of all small molecule B growth scores.

This corresponds to a Z-score cutoff value of +/−2.5. We identified any gene whose knockout exhibited a significant score when exposed to trimethoprim or sulfamethizole in the Nichols et al. dataset [28]. We then identified genes whose knockouts responded significantly across the majority of concentrations of both drugs. If these genes are transcribed as part of a polycistronic RNA, then a phenotype was considered significant if any mutant in that operon met the Z score requirement (|Z| > 2.5). For example, if *eck1864*Δ was significant at 1 trimethoprim concentration and *eck1865*Δ at another, the entire *ECK1864-66* operon was considered a significant hit at both those concentrations. For trimethoprim + sulfamethizole, this method identified 5 potential synergy prediction mutants/operons. We tested all small molecules predicted as synergistic for each of these synergy prediction mutants/operons, then calculated enrichment for successful predictions using a Fisher’s exact test. We also tested additional Z scores, |Z| > 1.96 and |Z| > 3.0, with no change in end result. |Z| > 3.0 identified *ECK1864-66*, *ECK0964*, and *ECK1710-13* as putative synergy prediction mutants. As shown in Figs 1 and S1, only *ECK1864-66* enriched for small molecules that synergize with trimethoprim. At |Z| > 1.96, *ECK4132-33*, *ECK1189*, and *ECK2901-04* were also identified as putative synergy prediction mutants. None of these mutants enriched for synergistic interactions with trimethoprim (S5 Fig).

### FICI calculations

We followed the same method as Hsieh et al. [92] with minor modifications. For MG1655, starting inoculation was 2 μl of an OD_600_ = 0.02 or 0.002 (10,000 or 1,000 cells per well of 200 μl medium, respectively). Any synergistic interaction was verified at both inoculation levels but the initial MG1655 screen using 10,000 cells per well. FICI assays for the MG1655 strain were incubated for 24 hours at 37°C. FICI assays of clinical strains were incubated for 12 hours at 37°C using an inoculum of 1,000 cells. OD_600_ was read on a BioTek plate reader model Synergy H1. Growth inhibition ≥90% compared to the no-drug control was considered significant.

### *E*. *coli* mutant construction

*E*. *coli* mutant *eck1864-66*::kanR was made in MG1655 by deleting the candidate gene or operon using the 1-step gene deletion method [93]. The putative knockout clones were confirmed by verification PCR with primers outside the deletion region. We amplified the *eck1864-66* knockout cassette with the following primers (gene-specific region in bold, kanamycin-specific region in regular typeface):

*eck1866*KO F:

**GTGAAGGATAAAGTGTATAAGCGTCCCGTTTCGATCTTAGTGGTCATCTA**TGTGTAGGCTGGAGCTGCTTCG

*eck1864*KO R:

**TTAACGCAGTCGCCCTCTCGCCAGGTTCAGCCGCGATTCGCTCATCTGCATC**CATATGAATATCCTCCTTAG

Colonies were selected on kanamycin. The clones were colony purified before PCR confirmation, then verified by PCR with the following primers:

ECK1864Ver F- CGACTCTCTGATGAGGCCTG

ECK1866Ver R-CCATTTACTATGACCTGCCA

### Microsource Spectrum library screen for trimethoprim’s synergistic partners

We inoculated either MG1655 wild-type or *eck1864-66*Δ cells at 1,000 cells per well (200 μl volume), then added either a vehicle control (DMSO) or small molecule to a final concentration of 10 μM per well. Plates were incubated for 18 hours at 37°C, then OD_600_ was measured on a plate reader. Small molecules that inhibited growth by more than 2.5-fold of the standard deviation of growth within each plate were considered significant.

### SOS pathway response assays

The plasmid with either GFP expressed under control of the *sulA* promoter or a promoterless plasmid containing only the GFP gene was transformed into MG1655 [94]. Strains were then grown to mid-log in MG1655 (OD_600_ approximately 0.4), then subcultured to OD_600_ to inoculate the experiment (2 ul into 200 μl M9 medium per well). We then performed a standard checkerboard assay, reading GFP signal (485 nm excitation and 528 nm emission) and OD_600_ at 0, 6, and 24 hours. The 24-hour timepoint determined the MIC90 and the difference between the 0- and 6-hour GFP signals determined the promoter activation.

### Bliss independence model of synergy

If a small molecule hit from the Microsource Spectrum library screen did not inhibit *E*. *coli* growth alone, we evaluated its synergistic interaction with trimethoprim by Bliss Independence [30] instead of checkerboard analysis. Briefly, we created a gradient of trimethoprim from 4 to 62.5 μg/ml, then added small molecules of interest at 10 μM or 100 μM. We calculated percent growth, then determined Bliss Independence by determining whether the growth inhibition caused by each small molecule alone is equal to the inhibition caused by combination treatment. If inhibition is greater in the combination, then the molecules act synergistically.

### Growth of *E*. *coli* mutants on trimethoprim, AZT, and rifampicin

Strains (S15 Table) were grown in M9 overnight, then diluted to an OD_66_ of 0.002 and inoculated into 96-well plates at the same density used for MIC and FICI assays. Each 96-well plate contained a gradient of either AZT, trimethoprim, or rifampicin, serially diluted in 2-fold increments. After 24 hours at 37°C, plates were measured in a BioTek Synergy H1. Each well was then normalized to the no-drug control for each mutant. Percent growth relative to this control is shown in the heat map. Each assay was repeated 3 times and the data averaged.

### Zebrafish (*Danio rerio*) husbandry

All zebrafish husbandry and experimental procedures were performed in accordance with the University of Utah and IACUC-approved protocols. Wildtype AB* zebrafish were maintained as breeding colonies on a 14-hour/10-hour light/dark cycle. Embryos were collected as mixed egg clutches and raised at 28.5°C in E3 medium (5 mM NaCl, 0.27 mM KCl, 0.4 mM CaCl2, 0.16 mM MgSO4; pH 7.4) containing 0.000016% methylene blue as an antifungal agent.

### Infection of zebrafish embryos

Embryos were anesthetized at 2 days post fertilization (dpf) with tricaine (0.77 mM ethyl 3-aminobenzoate methanesulfonate salt [Sigma-Aldrich]), embedded in 0.8% low-melt agarose without tricaine, and supplemented with E3 media lacking methylene blue. A bacteria culture of BEC8 or F11 was grown at 37°C overnight in 12 ml M9 minimal media. Prior to injection, 1 mL of culture at OD_600_ = 2.5 to 3.5 for BEC8 or OD_600_ = 1.7 was created. Filtered green food dye was added to the culture in a 1:10 dilution. 1 nL was injected into the pericardial cavity of embryos using an Olympus SZ61 stereomicroscope together with a YOU-1 micromanipulator (Narishige), a Narishige IM-200 microinjector, and a JUN-AIR model 3-compressor. Embryos were left to incubate at 28.5°C for 3 hours. Small molecules were mixed with green food dye, and 1 nL was injected into the yolk of embryos at 3 hpi. Dosages are listed in Table 4. (We estimated embryo mass based on Stehr et al. [95]). Additionally, drugs were supplemented to water 12 hours after yolk injections (15 hpi). Any embryos that were physically damaged during the procedures were discarded and excluded from further analysis. Embryos were unembedded and placed individually in a 96-well plate and left to incubate in 0.03% Instant Ocean at 28.5°C until we determined bacterial burden at 24 hpi for BEC8 or 19 hpi for F11. These timepoints result in comparable bacterial burden in the vehicle-treated embryos. Bacterial inoculation levels are shown in S4 Fig.

### Enumeration of bacterial numbers in zebrafish embryos

Embryos were euthanized at 24 (BEC8) or 19 (F11) hpi, then homogenized in 500 μL of PBS using a mechanical PRO 250 homogenizer (PRO Scientific). Homogenates were serially diluted and plated on LB agar plates (F11) or LB agar plates containing ampicillin (BEC8) and incubated overnight at 37°C. Any embryos dead and decaying by the euthanasia timepoint were excluded from further analysis, as survival curves show that dead embryos were rare at these timepoints [78] and were evenly distributed across treatment groups.

## Supporting information

S1 Fig Other putative synergy prediction mutants do not enrich for trimethoprim synergizers. O2M analysis identified five potential synergy response genes/operons. While a deletion of the operon *eck1864-44* enriched for trimethoprim and sulfamethizole synergizers (Fig 1), the other potential synergy prediction mutants did not. FICI scores are shown for (A) *eck0963—86*D, (B) *eck1082-86*D, (C) *eck1710-13*D, and (D) *eck3930*D. The color scheme is the same as in Fig 1: predicted synergistic molecules are labeled in green, known synergizers in purple, and negative control (predicted non-synergizers) in blue. The FICI cutoff for synergy is ≤ 0.5 (red line) and synergistic FICI values are marked with yellow bars on the graph. Non-synergistic values are colored blue. None of these mutants enrich for trimethoprim synergizers (p > 0.1 by Fisher’s exact test). The function of these genes/operons are: ECK0963-68: The hydrogenase 1 operon. ECK1082-86: Contains an amino pepidase, and oxidoreductase, and 2-octaprenyl-6-methoxyphenol hydroxylase, and a protein of unknown function. ECK1710-13: phenylalanine-tRNA synthetase subunits and transcriptional regulator. These gene functions do seem related to any of the functions of our new synergistic pairs. Individual FICI scores are the same as in Fig 1, since the putative synergy prediction mutants change the category of small molecules (e.g. from predicted synergizer to predicted non-synergizer and vice versa). Thus, the data for this figure are in S2 Table.
(TIF)

S2 Fig O2M analysis of vancomycin identifies synergistic interactions. Checkerboard results from vancomycin + predicted synergistic small molecules (green labels), known synergizer (purple label), and negative control small molecules (blue labels) that are not predicted to synergize with vancomycin. The FICI cutoff for synergy is ≤ 0.5 (red line) and synergistic FICI values are marked with yellow bars on the graph. Non-synergistic values are colored blue. FICI scores are shown in S16 Table.
(TIF)

S3 Fig Checkerboard data of clinical isolates. Heat map of growth (OD_600_) normalized to a no drug control. The yellow border represents the edge of growth, defined as less than 10% the cell density of the control. The left column contains all the drug combinations for blood isolate #7. The right column contains different clinical isolates treated with floxuridine + AZT. Blood isolate #4 and *K*. *pneumoniae* isolate #4 exhibit a synergistic response to this combination. Blood isolate #3 and *K*. *pneumoniae* isolate #1 do not exhibit a synergistic response. Data for this figure is in S17 Table.
(TIF)

S4 Fig Inoculation levels of zebrafish with *E*. *coli*. Post-inoculation, embryos are divided into either the experimental groups (Fig 7, ≥ 10 embryos per treatment per experiment) or the titering group. Embryos in the titer group (n ≥ 4 per experiment) were euthanized after inoculation, then homogenized and plated to LB + amp to determine their bacterial burden. Each datapoint represents a separate embryo. Inoculation levels are shown for each independent experimental replicates. (A) Inoculation levels for infections with MDR strain BEC8. (B) Inoculation levels for infections with drug-sensitive strain F11. Data for this figure is in S18 Table.
(TIF)

S5 Fig Additional putative synergy prediction mutants at |Z| > 1.96 cutoff do not enrich for synergistic interactions with trimethoprim. O2M analysis at a lower |Z| score identified three additional potential synergy response genes/operons. While a deletion of the operon *eck1864-44* enriched for trimethoprim and sulfamethizole synergizers (Fig 1), deletion of other potential synergy prediction mutants at |Z| > 2.5 (S1 Fig) and these mutants at |Z| > 1.96 did not. FICI scores are shown for (A) *eck1189*D, (B) *eck2901-04*D, and (C) *eck4132-33*D. The color scheme is the same as in Fig 1: predicted synergistic molecules are labeled in green, known synergizers in purple, and negative control (predicted non-synergizers) in blue. The FICI cutoff for synergy is ≤ 0.5 (red line) and synergistic FICI values are marked with yellow bars on the graph. Non-synergistic values are colored blue. None of these mutants enrich for molecules that act synergistically with trimethoprim. P-values were calculated using Fisher’s exact test. Similar to S1 Fig, the data for this figure is in S2 Table.
(TIF)

S1 Table Known synergistic antibiotics. (XLSX)

S2 Table FICI scores for trimethoprim. FICI scores were determined as described in Materials and Methods. The color scheme is the same as in Fig 1C: predicted synergizers are colored green, the positive control is colored purple, and predicted non-synergizers are colored blue. FICI ≤ 0.5 is considered synergistic.
(XLSX)

S3 Table FICI scores for sulfamethizole. FICI scores were determined as described in Materials and Methods. The color scheme is the same as in Fig 1D: predicted synergizers are colored green, the positive control is colored purple, and predicted non-synergizers are colored blue. FICI ≤ 0.5 is considered synergistic.
(XLSX)

S4 Table FICI scores of hits from synergizer screen. FICI scores were determined as described in Materials and Methods. FICI ≤ 0.5 is considered synergistic.
(XLSX)

S5 Table Bliss Independence scores of hits from synergizer screen. Bliss independence scores were calculated as described in Materials and Methods. Scores < 0 at both test concentrations were considered synergistic.
(XLSX)

S6 Table FICI scores for clinical isolates. FICI scores were determined as described in Materials and Methods. Data for trimethoprim + sulfamethizole and trimethoprim + AZT are shown. FICI ≤ 0.5 is considered synergistic.
(XLSX)

S7 Table sulA promoter induction levels. Data from Fig 4A.
(XLSX)

S8 Table Fluctuation analysis. Data from Fig 4B.
(XLSX)

S9 Table FICI scores for trimethoprim replacement molecules. FICI scores were determined as described in Materials and Methods. FICI ≤ 0.5 is considered synergistic.
(XLSX)

S10 Table Growth of mutants on AZT, trimethoprim, and rifampicin. Growth (expressed as % of cell density relative to no drug growth for each strain) for each mutant when grown in the presence of each small molecule.
(XLSX)

S11 Table FICI scores for trimethoprim substitutes against clinical isolates. FICI scores were determined as described in Materials and Methods. Data for hydroxyurea + AZT and floxuridine + AZT are shown. FICI ≤ 0.5 is considered synergistic.
(XLSX)

S12 Table Bacterial burden in zebrafish infection. Colony forming units (CFU) from each embryo infected in experiments in Figs 7 and S4.
(XLSX)

S13 Table FICI scores *E*. *coli* strain F11. FICI scores were determined as described in Materials and Methods. Growth was measured at 12 hours post-inoculation.
(XLSX)

S14 Table MIC values determined with and without human serum. The MICs of AZT, floxuridine, trimethoprim, and sulfamethizole were tested with strains F11 and BEC8 in both M9 media or M9 media containing 20% Human AB serum. There is no data for trimethoprim and sulfamethizole for BEC8 due to drug resistance, which led to difficulties achieving MIC. Our data show little difference in the MICs of AZT and floxuridine with and without serum. This is supported by the literature, which shows less than 38% of AZT and 8–12% of fluorouracil (the active compound of floxuridine) binding to proteins [96]. We similar or slightly increased MICs for trimethoprim and sulfamethizole with and without serum. Literature shows 40–70% or trimethoprim [97] and 85–90% of sulfamethizole [98] binds to proteins, which support this observation [96].
(XLSX)

S15 Table Strains used in this study. (XLSX)

S16 Table FICI scores for vancomycin. FICI scores were determined as described in Materials and Methods. The color scheme is the same as in S2 Fig: predicted synergizers are colored green, the positive control is colored purple, and predicted non-synergizers are colored blue. FICI ≤ 0.5 is considered synergistic.
(XLSX)

S17 Table Bacterial inoculation of zebrafish. Zebrafish embryos were euthanized immediately after infection (as described in Materials and methods) to determine starting bacterial burden.
(XLSX)

S18 Table Raw data from S3 Fig. (XLSX)

S1 Text Step-by-step instructions on how to perform O2M analysis. (DOCX)

## Abbreviations

AZT: azidothymidine
CFU: colony-forming unit
dATP: deoxyadenosine triphosphate
dcd: deoxycytidine deaminase
dCTP: deoxycytidine triphosphate
dNTP: deoxynucleotide triphosphate
dTTP: deoxythymidine triphosphate
FICI: fractional inhibitory concentration index
hpi: hours postinoculation
HU: hydroxyurea
LB: lysogeny broth
MDR: multidrug-resistant
MIC: minimum inhibitory concentration
ndk: nucleoside diphosphate kinase
O2M: overlap^2^ method
UTI: urinary tract infection
