# Supplementary figures and images for: High-throughput identification and rational design of synergistic small-molecule pairs for combating and bypassing antibiotic resistance
Source: PLoS Biol. 2017 Jun 20;15(6):e2001644. doi: 10.1371/journal.pbio.2001644 (PMC5478098; doi:10.1371/journal.pbio.2001644)

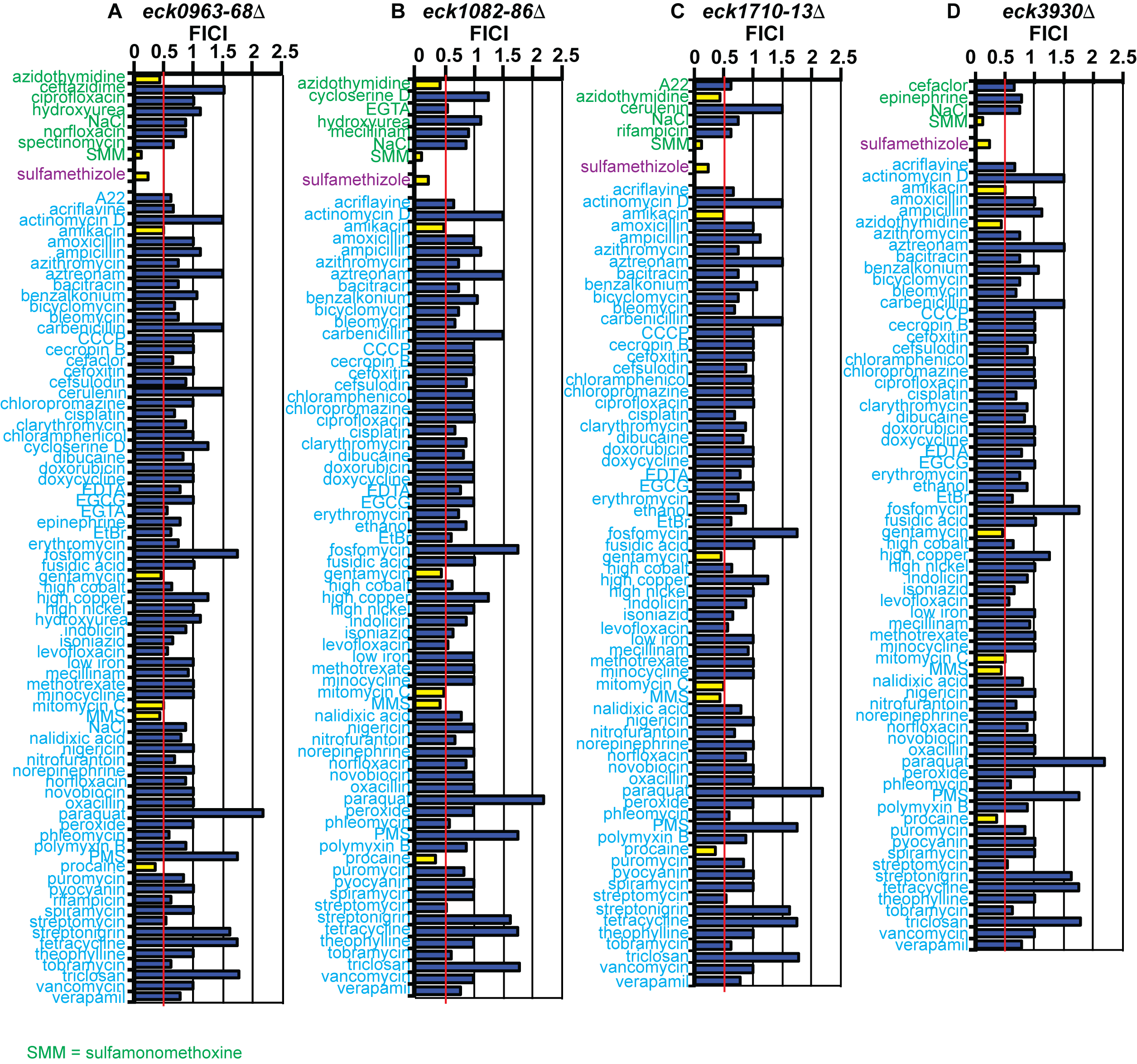

Supplement: S1 Fig — O2M analysis identified five potential synergy response genes/operons. While a deletion of the operon eck1864-44 enriched for trimethoprim and sulfamethizole synergizers (Fig 1), the other potential synergy prediction mutants did not. FICI scores are shown for (A) eck0963—86D, (B) eck1082-86D, (C) eck1710-13D, and (D) eck3930D. The color scheme is the same as in Fig 1: predicted synergistic molecules are labeled in green, known synergizers in purple, and negative control (predicted non-synergizers) in blue. The FICI cutoff for synergy is ≤ 0.5 (red line) and synergistic FICI values are marked with yellow bars on the graph. Non-synergistic values are colored blue. None of these mutants enrich for trimethoprim synergizers (p > 0.1 by Fisher’s exact test). The function of these genes/operons are: ECK0963-68: The hydrogenase 1 operon. ECK1082-86: Contains an amino pepidase, and oxidoreductase, and 2-octaprenyl-6-methoxyphenol hydroxylase, and a protein of unknown function. ECK1710-13: phenylalanine-tRNA synthetase subunits and transcriptional regulator. These gene functions do seem related to any of the functions of our new synergistic pairs. Individual FICI scores are the same as in Fig 1, since the putative synergy prediction mutants change the category of small molecules (e.g. from predicted synergizer to predicted non-synergizer and vice versa). Thus, the data for this figure are in S2 Table. (TIF) [file pbio.2001644.s001.tif]

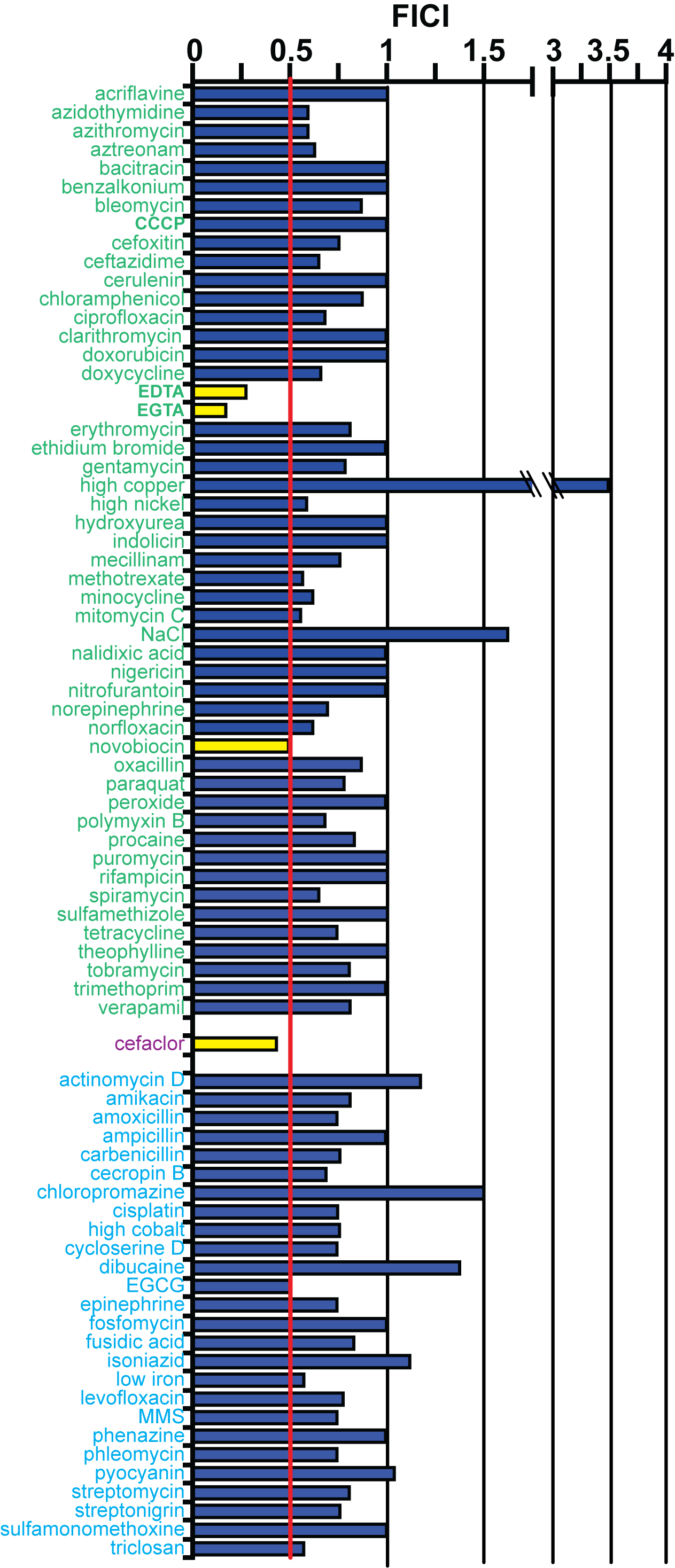

Supplement: S2 Fig — Checkerboard results from vancomycin + predicted synergistic small molecules (green labels), known synergizer (purple label), and negative control small molecules (blue labels) that are not predicted to synergize with vancomycin. The FICI cutoff for synergy is ≤ 0.5 (red line) and synergistic FICI values are marked with yellow bars on the graph. Non-synergistic values are colored blue. FICI scores are shown in S16 Table. (TIF) [file pbio.2001644.s002.tif]

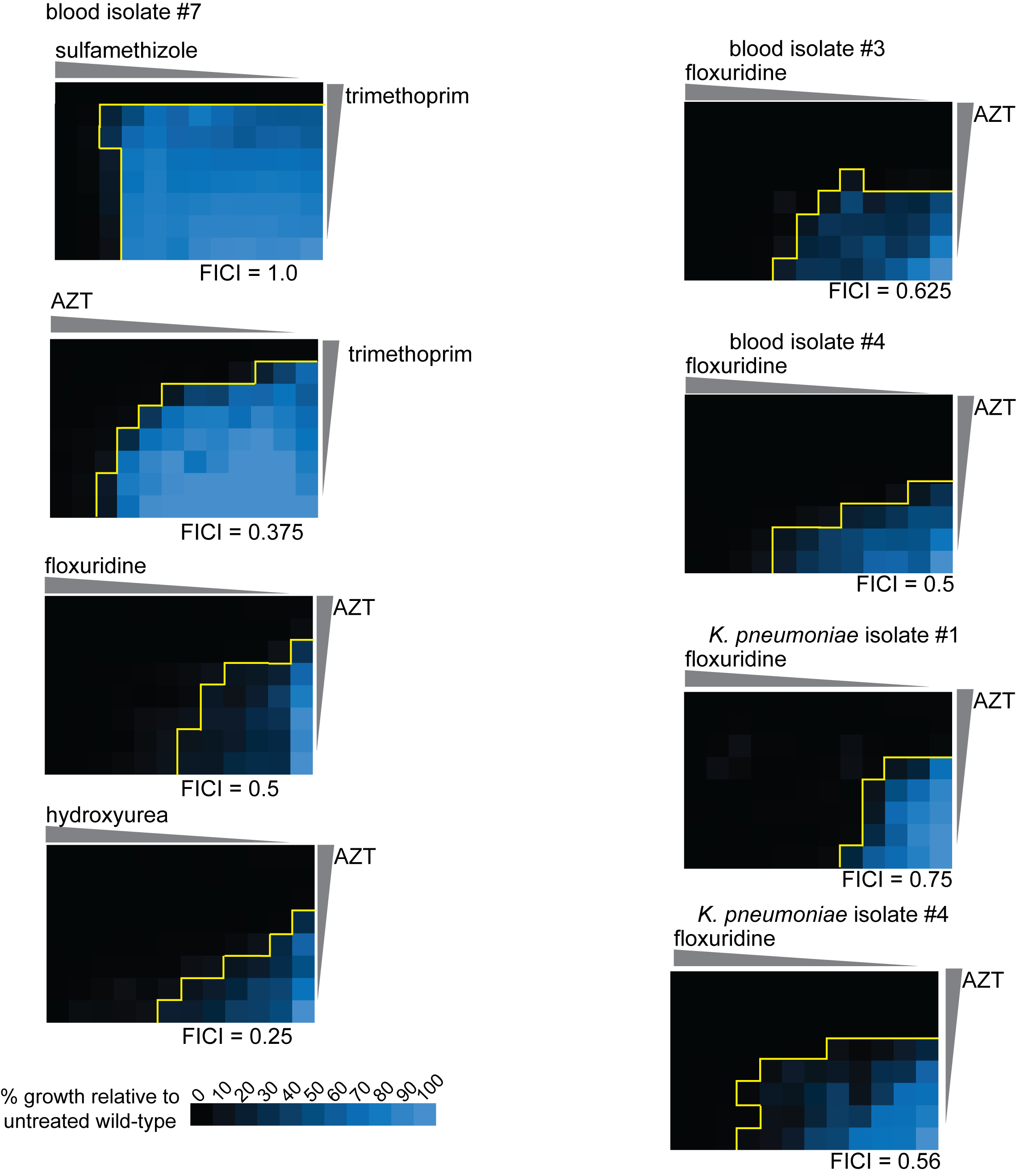

Supplement: S3 Fig — Heat map of growth (OD600) normalized to a no drug control. The yellow border represents the edge of growth, defined as less than 10% the cell density of the control. The left column contains all the drug combinations for blood isolate #7. The right column contains different clinical isolates treated with floxuridine + AZT. Blood isolate #4 and K. pneumoniae isolate #4 exhibit a synergistic response to this combination. Blood isolate #3 and K. pneumoniae isolate #1 do not exhibit a synergistic response. Data for this figure is in S17 Table. (TIF) [file pbio.2001644.s003.tif]

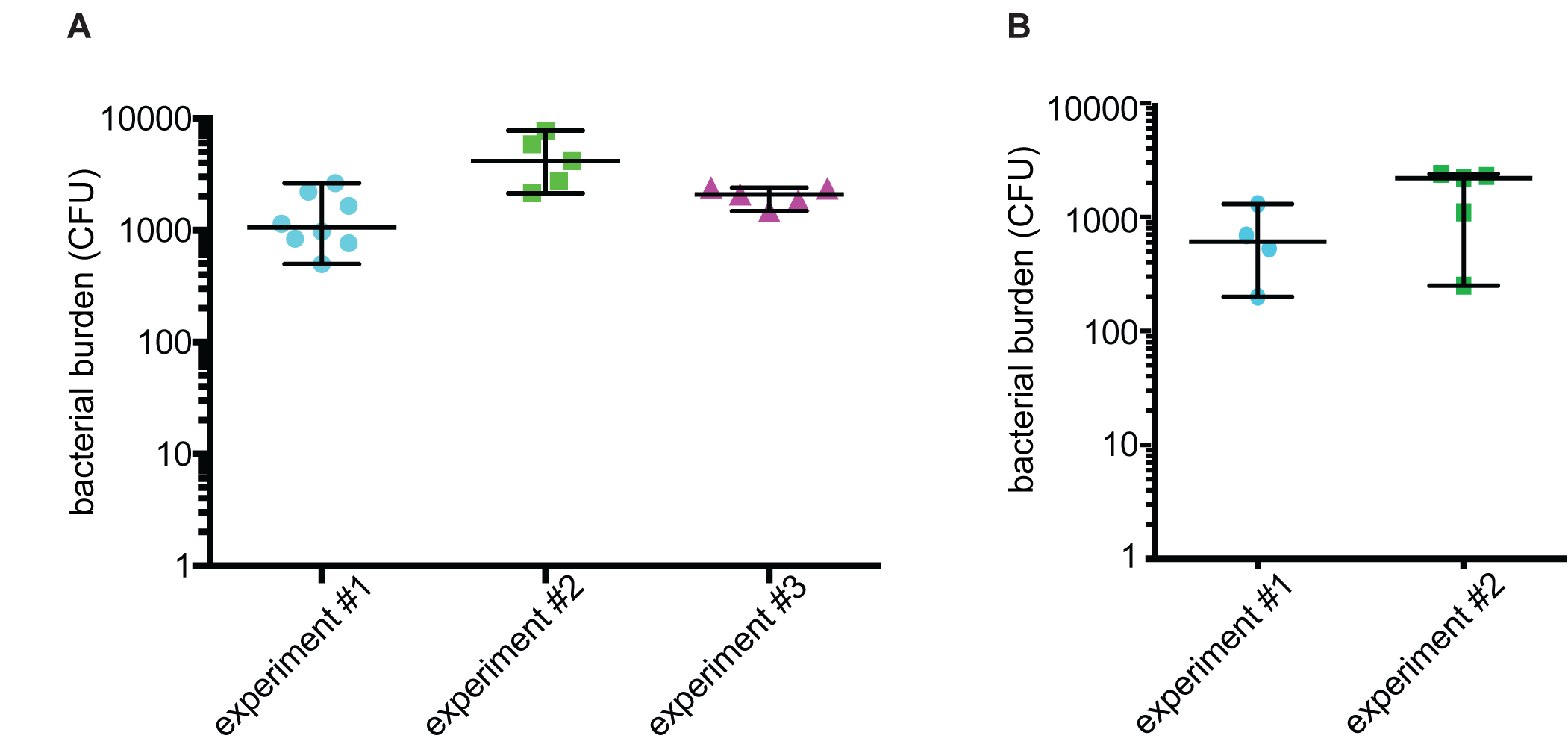

Supplement: S4 Fig — Post-inoculation, embryos are divided into either the experimental groups (Fig 7, ≥ 10 embryos per treatment per experiment) or the titering group. Embryos in the titer group (n ≥ 4 per experiment) were euthanized after inoculation, then homogenized and plated to LB + amp to determine their bacterial burden. Each datapoint represents a separate embryo. Inoculation levels are shown for each independent experimental replicates. (A) Inoculation levels for infections with MDR strain BEC8. (B) Inoculation levels for infections with drug-sensitive strain F11. Data for this figure is in S18 Table. (TIF) [file pbio.2001644.s004.tif]

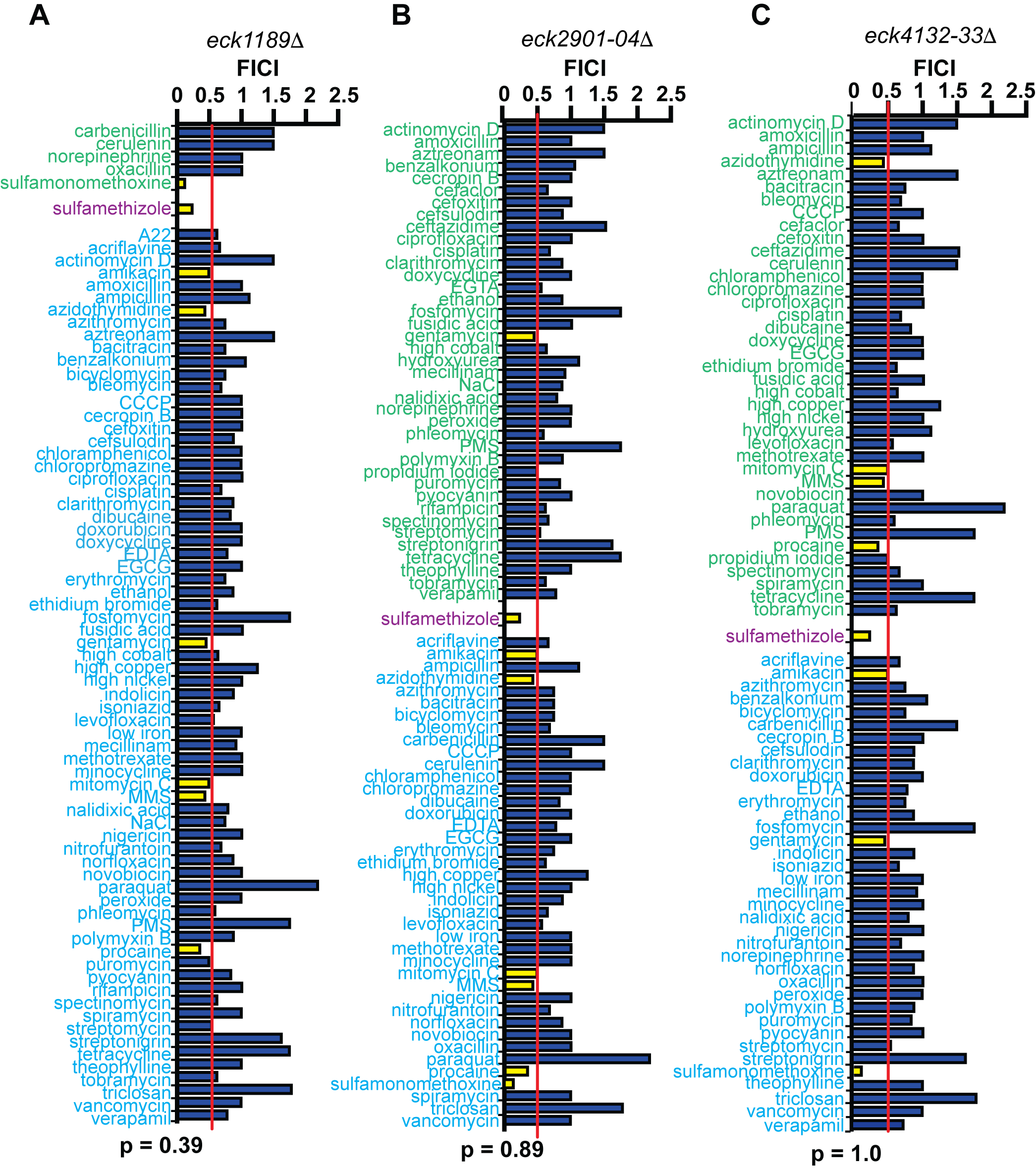

Supplement: S5 Fig — O2M analysis at a lower |Z| score identified three additional potential synergy response genes/operons. While a deletion of the operon eck1864-44 enriched for trimethoprim and sulfamethizole synergizers (Fig 1), deletion of other potential synergy prediction mutants at |Z| > 2.5 (S1 Fig) and these mutants at |Z| > 1.96 did not. FICI scores are shown for (A) eck1189D, (B) eck2901-04D, and (C) eck4132-33D. The color scheme is the same as in Fig 1: predicted synergistic molecules are labeled in green, known synergizers in purple, and negative control (predicted non-synergizers) in blue. The FICI cutoff for synergy is ≤ 0.5 (red line) and synergistic FICI values are marked with yellow bars on the graph. Non-synergistic values are colored blue. None of these mutants enrich for molecules that act synergistically with trimethoprim. P-values were calculated using Fisher’s exact test. Similar to S1 Fig, the data for this figure is in S2 Table. (TIF) [file pbio.2001644.s005.tif]
