# Supplementary material for: High-throughput identification and rational design of synergistic small-molecule pairs for combating and bypassing antibiotic resistance
Source: PLoS Biol. 2017 Jun 20;15(6):e2001644. doi: 10.1371/journal.pbio.2001644 (PMC5478098; doi:10.1371/journal.pbio.2001644)
Supplement: S1 Text — (DOCX) [file pbio.2001644.s024.docx]

O2M analysis of chemical genetics data

The following is performed using the dataset from Nichols *et al.* [1]. This analysis is based on the one published in Brown *et al.* [2]. If users are working with a different file, we recommend adjusting the format so that it matches this file.

1. Open the file in Excel or a preferred spreadsheet program. The small molecule and conditions are along the x-axis (in columns, with the labels in row 2) and the genes are listed on the y-axis (in rows, with the labels in column A). Each number represents a growth score that quantitatively describes the interaction between a particular mutant and a chemical condition. Negative scores are more sensitive than average and positive scores more resistant.
2. Calculate the mean and standard deviation of growth for each genetic mutant (e.g. in column LO) and for each small molecule/condition.
3. Calculate the desired Z scores. We commonly use |Z| > 2.5 or |Z| > 3.0. Calculate both negative scores [Z(2.5) = mean – 2.5*standard deviation] and positive scores [Z(2.5) = mean + 2.5*standard deviation]. Growth scores are significant is the positive scores are larger than the positive Z score and the negative scores smaller than the negative Z score. Growth scores between the two Z scores are not significant.
4. Open a new sheet. Copy the gene labels (column A), data for small molecules of interest (e.g. trimethoprim and sulfamethizole and multiple concentrations), and corresponding Z score ranges into a new sheet. Use the “paste special function” and paste values only.
5. Identify genes whose mutants show a significant growth score on the small molecules of interest. This can be done several ways:
   1. Sort each column (and corresponding gene labels) by values. Make a list of values and gene mutants that are smaller or larger than the Z score cutoff.
   2. Use an “IF” function to automatically calculate if each growth score is significant. We usually set it up so that if the score is significant, the output is a number, and if it is not, the output is “false.”
6. Make a list of significant gene mutants (with growth score) for each small molecule and concentration.
7. Determine if the genes are part of an operon. This data is easily available at Ecoliwiki.net. On each gene page, look under the “Regulation/expression” category. This will list “transcriptional unit.” Click on the link and determine if the transcriptional unit consists of multiple genes.
8. Return to the list of significant gene mutants for each small molecule of interest. Compare each concentration of a single drug and determine how many times each gene is listed as significant. A hit to a gene in an operon counts as a hit to the entire operon. For example, if gene #1 and gene #2 are in the same transcription unit, and if gene #1 is listed under concentration #1 and gene #2 is listed under concentration #2, then we count this as two counts of significance (credited to the operon) rather than one count of significance for each gene.
9. Identify the list of genes/operons whose mutants show a significant response on a majority of concentrations of each small molecule of interest.
10. Compare the significant gene/operon list between two known synergistic small molecules.
11. Identify genes/operons listed under both small molecules. These are the putative synergy prediction mutants.
12. Return to the sheet with the full dataset. Extract and copy all the growth scores for each putative synergy prediction mutant/operon to a new sheet. Remember to copy Z score data and labels for the small molecule conditions.
13. Identify the small molecules that induce a significant response from each putative synergy prediction mutant. If the prediction mutant is part of an operon, perform these steps for all mutants corresponding to each gene in that operon.
    1. Significant responses can be identified by either sorting by value or by using the “IF” function. See step 5.
14. List each small molecule/condition with its corresponding synergy prediction mutant. These are the molecules/conditions that we predict would act synergistically with the starting small molecules. At this stage, we do not require that a small molecule appear in the list at multiple concentrations; if it appears at least once, we call it a “predicted synergizer.”
15. If some of the synergy prediction mutants are part of operons, any molecule/condition identified by any gene/mutant in the operon is considered a predicted synergizer.
16. Any small molecules/conditions that do not induce a significant growth score from a synergy prediction mutant is considered a “predicted non-synergizer.”
17. This analysis should produce a list of “predicted synergizer” small molecules and “predicted non-synergizer” small molecules for each synergy prediction mutant.
18. Test each small molecule/condition in step #17 in the appropriate assay (e.g. checkerboard assays or Bliss independence).
19. To determine if a putative synergy prediction mutant indeed enriches for synergistic interactions, create a contingency table:

|  | Predicted synergizers | Predicted non-synergizers |
| --- | --- | --- |
| # synergistic in experiments |  |  |
| # not synergistic in experiments |  |  |

1. Calculate significance using Fisher’s exact test. We recommend the following online calculator: <http://graphpad.com/quickcalcs/contingency1.cfm>
2. If the p value is significant, than the putative synergy prediction mutant successfully enriches for synergistic interactions.

**References**

1. Nichols RJ, Sen S, Choo YJ, Beltrao P, Zietek M, Chaba R*, et al.* Phenotypic Landscape of a Bacterial Cell. Cell. 2010;144(1):143-56.

2. Brown JCS, Nelson J, VanderSluis B, Deshpande R, Butts A, Kagan S*, et al.* Unraveling the biology of a fungal meningitis pathogen using chemical genetics. Cell. 2014;159(5):1168-87.
